# Entrectinib attenuates LPS-induced neuroinflammation by inhibiting JNK, p38, and AKT pathways and ameliorates cognitive impairment

**DOI:** 10.1007/s12272-026-01608-x

**Published:** 2026-04-05

**Authors:** Hanwoong Woo, Sung Wook Kim, Sohee Kim, Sehyun Chae, Jieun Kim

**Affiliations:** 1 Multidimensional Genomics Research Center, Kangwon National University, Chuncheon, 24341 Republic of Korea; 2 Department of Neurovascular Unit Research Group, Korea Brain Research Institute (KBRI), 61, Cheomdan-ro, Dong-gu, Daegu, 41062 Republic of Korea; 3 Department of Bio-Health Convergence, Kangwon National University, Chuncheon, 24341 Republic of Korea; 4 Division of Chemical Engineering and Bioengineering, College of Art Culture and Engineering, Kangwon National University, Chuncheon, 24341 Republic of Korea; 5 Department of Bio-Health Technology, College of Biomedical Science, Kangwon National University, Chuncheon, 24341 Republic of Korea

**Keywords:** Entrectinib, Neuroinflammation, Memory, Microglia, Phagocytosis

## Abstract

Entrectinib, a Food and Drug Administration-approved medication for cancers such as non-small cell lung cancer, inhibits tropomyosin receptor kinases and penetrates the blood–brain barrier. Despite its approval, the effects of Entrectinib on neuroinflammatory responses and cognitive function within the central nervous system remain unclear. This study demonstrates that Entrectinib modulates lipopolysaccharide (LPS)-induced pro- and anti-inflammatory factors by suppressing JNK, p38, and AKT signaling, as well as NF-κB and STAT3 activity, in primary microglia. Entrectinib reduced CD16/32 levels, increased CD206 expression, enhanced phagocytic activity, and upregulated receptors and cytoskeletal genes in vitro. Additionally, Entrectinib decreased proinflammatory cytokines and inhibited JNK/p38/AKT and NF-κB/STAT3 signaling in the hippocampus of LPS-treated mice. Notably, Entrectinib ameliorated LPS-induced memory impairments in vivo. Collectively, these findings indicate that Entrectinib attenuates neuroinflammation and improves memory performance, supporting its potential therapeutic relevance for neuroinflammation-associated cognitive disorders.

**Supplementary Information:**

The online version contains supplementary material available at 10.1007/s12272-026-01608-x.

## Introduction

Entrectinib is an FDA-approved anti-cancer medication used to treat neurotrophic tyrosine receptor kinase fusion-positive cancers (Kita et al. 2017; Suzuki et al. 2022; Chen et al. 2024). Entrectinib antagonizes tropomyosin receptor kinase (TRK) A, B, and C, as well as other receptor tyrosine kinases (Jiang et al. 2021). Recent studies indicate that Entrectinib selectively inhibits TRKs at low concentrations and demonstrates high penetration across the blood–brain barrier (Fischer et al. 2020; Desai et al. 2022). Previous work has established the efficacy of Entrectinib in non-small cell lung cancer (NSCLC); however, its role in regulating neuroinflammatory processes within the central nervous system (CNS) remains unclear.

TRKs are a family of tyrosine kinase receptors with affinity for neurotrophins, including nerve growth factor (NGF), brain-derived neurotrophic factor (BDNF), and neurotrophin-3 (NT-3) (Huang and Reichardt 2001). These receptors are essential for neuronal differentiation, survival, synaptic function, and plasticity (Longo and Massa 2013). Beyond their developmental roles, TRK signaling contributes to glial reactivation during CNS neuroinflammation (Qian et al. 2007; Ding et al. 2020). For instance, neuronal BDNF release activates glial TRK-B and promotes proinflammatory cytokine production via p38 and JNK signaling in cystitis-induced conditions (Ding et al. 2020). Despite this evidence, whether TRK inhibition by Entrectinib modulates neuroinflammatory responses and alters microglial functional states remains uncertain.

Neuroinflammation refers to an inflammatory response within the CNS driven by a network of cytokines, chemokines, reactive oxygen species, and secondary messengers produced by microglia (DiSabato et al. 2016). Microglia are critical regulators of CNS homeostasis and exhibit diverse phenotypes with neurotoxic or neuroprotective properties (DiSabato et al. 2016). During the neurotoxic phase, often described as reactive microglia, activation is triggered by pathogens or proinflammatory mediators such as lipopolysaccharide (LPS) and interferon gamma (IFN-γ). In this state, microglia release proinflammatory factors, including tumor necrosis factor (TNF)-α, interleukin (IL)-6, IL-1β, and inducible nitric oxide synthase (iNOS), which promote oxidative stress in response to immune stimuli (Ransohoff 2016). Conversely, anti-inflammatory microglia secrete mediators such as transforming growth factor-β, IL-10, IL-4, and IL-1R, reflecting tissue-protective and neuronal repair functions within the brain (Ransohoff 2016). Recent evidence indicates that regulation of reactive microglial states is essential for maintaining microglial homeostasis during CNS neuroinflammation (Ransohoff 2016).

Despite the established role of TRK signaling in microglial neuroinflammatory responses, the effects of Entrectinib on TRK-related neuroinflammation have not been examined. Accordingly, this study investigated the impact of Entrectinib on LPS-induced neuroinflammatory responses in primary microglia and LPS-treated mice.

This study, therefore, examined whether post-treatment with Entrectinib influences TRK phosphorylation in LPS-treated primary microglia and the hippocampus. We further explored the potential involvement of neuroinflammation-related signaling pathways, including JNK, p38, and AKT, as well as NF-κB and STAT3 activity. In addition, the study assessed whether Entrectinib is associated with changes in microglial phenotypic markers, including those related to reactive and anti-inflammatory states, both in vitro and in vivo. The potential effects of Entrectinib on amyloid beta (Aβ) phagocytosis and related gene expression in primary microglia were also investigated. Finally, we examined whether treatment with Entrectinib following LPS administration is associated with alterations in short-term and long-term memory performance in mice, with the aim of clarifying its therapeutic relevance for neuroinflammation-related microglial dysfunction and cognitive decline.

## Materials and methods

### Ethics statement

Animal experiments were conducted in accordance with the guidelines approved by the Institutional Animal Care and Use Committees (IACUC) of Kangwon National University (KW-230907-3).

### Entrectinib and LPS treatments

For in vitro experiments, LPS from *Escherichia coli* (Sigma, Cat. No. L2630, St. Louis, MO, USA) was dissolved in phosphate-buffered saline (PBS), and primary microglial cells were exposed to LPS at a concentration of 200 ng/mL. For in vivo studies, mice received intraperitoneal (i.p.) injections of LPS (250 μg/kg or 1 mg/kg) once daily for 8 or 16 days to induce an inflammatory response.

Entrectinib (Selleckchem, Cat. No. S7998, Houston, TX, USA) was dissolved in dimethyl sulfoxide (DMSO) for in vitro treatments and applied to primary microglial cells at a concentration of 1 μM unless otherwise specified. For in vivo experiments, the vehicle solution consisted of 5% DMSO, 40% PEG, and 5% Tween 80 in deionized water. Mice were administered Entrectinib at a dose of 10 mg/kg.

### Primary microglia culture

Primary microglial cells were isolated from the brains of C57BL/6 J mice on postnatal day 1. The cerebrum was dissected and placed in ice-cold Hanks’ balanced salt solution. After two washes with low-glucose Dulbecco’s Modified Eagle Medium (DMEM), the brain tissue was passed through a 70-μm nylon mesh and then cultured in low-glucose DMEM supplemented with 10% FBS, 100 U/mL penicillin, and 100 μg/mL streptomycin in a 5% CO₂ incubator. On day 14, cells underwent mild trypsinization using 0.25% trypsin–EDTA in low-glucose DMEM supplemented with additional EDTA and CaCl_2_. After washing to eliminate the astrocyte layer, primary microglia were detached using 0.25% trypsin–EDTA and centrifuged twice at 680 × *g* for 10 min before use.

### BV2 microglial cells

The BV2 microglial cell line was obtained from Koram Bio-Tech (Seoul, Republic of Korea). Cells were maintained in high-glucose DMEM (Cytiva, Cat. No. SH30243.01, Wilmington, DE, USA) supplemented with 5% FBS (Gibco, Cat. No. 16000–044, Waltham, MA, USA), 100 U/mL penicillin G, and 100 μg/mL streptomycin, and incubated at 37 °C in a humidified atmosphere containing 5% CO₂.

### TRK siRNA transfection

Mouse primary microglial cells were transfected with siRNAs targeting TRK-A or TRK-B (Dharmacon; Cat. Nos. L-049564-00 and L-048017-00, respectively; Lafayette, CO, USA) or with a non-targeting scrambled siRNA as a control (Dharmacon; Cat. No. D-001810–01). Each siRNA was prepared as a 10 μM stock solution and diluted in serum-free medium. Diluted siRNA was gently mixed with diluted DharmaFECT transfection reagent (Dharmacon; Cat. No. T-2003-03) according to the manufacturer’s instructions. The growth medium was replaced with antibiotic-free complete medium, and the transfection mixture was added to each well to achieve a final siRNA concentration of 50 nM. Cells were incubated for 48 h at 37 °C in a humidified atmosphere containing 5% CO₂. Following transfection, cells were treated with LPS and/or Entrectinib as indicated for subsequent analyses.

### CCK-8 assay

Cells were plated in 96-well plates at a density of 1 × 10^4^ cells per well and incubated in FBS-free medium for 1 h. Cells were then treated with Entrectinib at concentrations of 0.1, 1, 5, or 10 μM, or with 1% DMSO as a vehicle control, for 12 or 24 h. Following treatment, 10% CCK-8 solution (Dojindo, Cat. No. CK04, Kumamoto, Japan) was added to the FBS-free medium, and cells were incubated for an additional 2 h. Absorbance at 450 nm was measured using a Multiskan Skyhigh microplate spectrophotometer (Thermo Scientific, Waltham, MA, USA) to assess cell viability.

### Real-time polymerase chain reaction (PCR)

The mRNA levels of genes associated with inflammatory responses (*Il1β, Il6, Tnfα, Inos, Ccl2, Il23α, Il13, and Il4*), phagocytosis (*Trem2, Sorl1, Cd33, and Cr2*), and cytoskeletal regulation (*Vav1* and *Cdc42*) in primary microglial cells or hippocampal tissue were quantified using real-time PCR. RNA was extracted from primary microglia or hippocampal tissue using Nucleozol (Macherey-Nagel, Cat. No. 740404.200, Dueren, Germany) according to the manufacturer’s instructions. Complementary DNA (cDNA) was synthesized using the Superscript cDNA Premix Kit II (GeNetBio, Cat. No. SR-5000, Daejeon, Republic of Korea), and real-time PCR was performed for 40 cycles using the SensiFAST™ SYBR® No-ROX Kit (Bioline, Cat. No. BIO-98050, Memphis, TN, USA) on a CFX Duet Real-Time PCR System (Bio-Rad, Hercules, CA, USA). *Gapdh* cycle threshold (Ct) values were used for normalization. Fold changes in LPS-or LPS + Entrectinib-treated cells or tissue were calculated relative to the vehicle-treated control. Primer sequences are listed in Table S1.

### Cytosolic and nuclear fractionation

Nuclear levels of phosphorylated NF-κB (p-NF-κB) and phosphorylated STAT3 (p-STAT3) were examined in primary microglia treated with 200 ng/mL LPS or PBS for 30 min, followed by exposure to 1 μM Entrectinib or 1% DMSO as a vehicle for 5.5 h. Cells were resuspended in cytosolic fractionation buffer containing 10 mM HEPES (pH 7.4), 10 mM KCl, and 0.05% NP-40 and incubated on ice for 20 min. The lysates were then centrifuged at 14,000 rpm for 10 min at 4 °C. The resulting supernatant, corresponding to the cytosolic fraction, was transferred to a clean tube. Radioimmunoprecipitation assay (RIPA) lysis buffer (Thermo Scientific, Cat. No. 89901, Waltham, MA, USA) was added to the remaining pellet, which was subsequently sonicated to disrupt nuclear components. After incubation on ice for an additional 10 min, the nuclear lysate was centrifuged again at 14,000 rpm for 10 min at 4 °C. The resulting nuclear fraction was used for western blot analysis to assess nuclear p-NF-κB and p-STAT3 levels.

### Western blotting

Western blotting was performed on primary microglia and hippocampal tissue treated with LPS or PBS, followed by Entrectinib or DMSO as a vehicle. Samples were lysed using RIPA lysis buffer and sonicated prior to centrifugation at 12,000 rpm for 10 min. Protein concentrations in the supernatants were quantified relative to a standard BSA solution, and 20 μg of protein was loaded onto 8% or 10% SDS-PAGE gels according to experimental requirements. Proteins were transferred to a nitrocellulose membrane (Amersham™ Protran™ 0.2 µm NC, Cytiva, Cat. No. 10600001, Wilmington, DE, USA), blocked with either 5% skim milk or 5% BSA at room temperature for 1 h, and incubated overnight at 4 °C with the following primary antibodies: anti-p-TRK anti-TRK (pan), anti-p-JNK, anti-JNK, anti-p-p38, anti-p38, anti-p-AKT, anti-AKT, anti-p-STAT3, anti-STAT3, anti-p-NF-κB, anti-NF-κB, anti-histone, anti-GAPDH, anti-PSD95, and anti-SYP. Membranes were then incubated with HRP-conjugated goat anti-mouse IgG (1:10,000; Invitrogen, Cat. No. 31430, Carlsbad, CA, USA) or goat anti-rabbit IgG (1:10,000; Promega, Cat. No. W4011, Madison, WI, USA) for 1 h. Protein detection was performed using the ECL™ Prime Western Blotting System (Cytiva, Cat. No. RPN2232, Wilmington, DE, USA). For reprobing, membranes were treated with Restore™ Western Blot Stripping Buffer (Thermo Scientific, Cat. No. 21059, Waltham, MA, USA). Images were acquired and analyzed using the ChemiDoc MP™ Imaging System (Bio-Rad, Hercules, CA, USA). Antibody catalog numbers and dilution factors are provided in Table S2.

### Immunocytochemistry

For immunocytochemistry, cells were fixed with 4% paraformaldehyde for 10 min and washed three times with PBS. Cells were then incubated overnight with the following primary antibodies diluted in PBS containing FBS: anti-p-TRK (pan), anti-p-JNK, anti-p-p38, anti-p-AKT, anti-p-STAT3, anti-p-NF-κB, anti-CD11b, anti-CD16/32, anti-CD206, and anti-Iba1 (detailed information is provided in Table S2). After incubation, cells were washed with PBS and treated with Alexa Fluor 488- (1:200, Invitrogen, Cat. No. A11006, Carlsbad, CA, USA), 594- (1:200, Invitrogen, Cat. No. A11012, Carlsbad, CA, USA), or 647- (1:200, Jackson ImmunoResearch, Cat. No. 103-605-155, Cambridge, UK) conjugated secondary antibodies for 2 h at room temperature. Cells were subsequently washed three times with PBS and incubated with DAPI solution (Thermo Scientific, Cat. No. 62248, Waltham, MA, USA) for 10 min. Following nuclear staining, cells were washed and mounted using fluorescence mounting medium (Dako, Cat. No. S3023, Santa Clara, CA, USA). Images were acquired using an A1/Ni-E microscope (Nikon, Tokyo, Japan) and analyzed with ImageJ software (US National Institutes of Health, Bethesda, MD, USA).

### Animals and immunofluorescence staining

Animal experiments were conducted in accordance with the guidelines approved by the Institutional Animal Care and Use Committees (IACUC) of Kangwon National University (KW-230907-3). Male C57BL/6 J mice (8 weeks old; Orient-Bio Company, Gyeonggi-do, Korea) were housed under controlled conditions (22 ± 2 °C, 50 ± 5% humidity) on a 12-h light/dark cycle in a pathogen-free environment, with ad libitum access to food and water. Mice were randomly assigned to three groups: control, LPS, and LPS + Entrectinib. For in vivo pre-treatment, mice received daily intraperitoneal injections of Entrectinib (10 mg/kg) or vehicle (5% DMSO, 40% PEG, 5% Tween 80 in deionized water) for 8 or 16 days, depending on group assignment. Thirty minutes after Entrectinib administration, mice were injected intraperitoneally with 250 μg/kg LPS or PBS. For post-treatment experiments, mice received intraperitoneal injections of LPS (1 mg/kg) or PBS, followed by administration of Entrectinib (10 mg/kg) or vehicle 30 min later. Following behavioral testing, mice were perfused with PBS and fixed with 4% paraformaldehyde. Brains were post-fixed in 4% paraformaldehyde at 4 °C for 24 h, cryoprotected in 30% sucrose in PBS for 72 h, and sectioned into 30-μm slices using a cryostat microtome (Leica CM1850, Wetzlar, Germany). Sections were blocked with 5% normal goat serum (Vector Laboratories, Burlingame, CA, USA) for 2 h at room temperature and immunostained overnight at 4 °C with the following primary antibodies: anti-p-TRK, anti-CD11b, anti-p-NF-κB, anti-p-STAT3, anti-CD16/32, and anti-CD206 (antibody details are provided in Table S2). Sections were washed with PBST buffer and incubated with Alexa 555- (1:200, Invitrogen, Cat. No. A21434, Carlsbad, CA, USA), Alexa 594- (1:200, Invitrogen, Cat. No. A11012, Carlsbad, CA, USA), or Alexa 488-conjugated secondary antibodies (1:200, Invitrogen, Cat. No. A11008, A11006, Carlsbad, CA, USA) for 2 h at room temperature. After additional washing with PBST buffer, sections were incubated with DAPI solution for 10 min. Following nuclear staining, sections were mounted on glass slides using VectaShield mounting medium for fluorescence (Vector Laboratories, Cat. No. H-1000, Burlingame, CA, USA). Fluorescence images were acquired using a DMi8 microscope (Leica Microsystems, Wetzlar, Germany) and analyzed with ImageJ software.

### Phagocytosis assay and live-cell imaging

The phagocytic activity of BV2 microglial cells was evaluated by assessing Aβ clearance. Prior to Aβ exposure, BV2 microglial cells were pre-treated with 200 ng/mL LPS or PBS for 30 min, followed by treatment with 1 μM Entrectinib or 1% DMSO as a vehicle for 23.5 h. During this period, 250 nM Alexa Fluor 488–conjugated Aβ1-42 (Anaspec, Cat. No. AS-60479-01, Fremont, CA, USA) was incubated in serum-free medium at 37 °C for 24 h. BV2 microglial cells were then exposed to the pre-incubated Aβ1-42 for 1 h. Cells were subsequently fixed with 4% paraformaldehyde for 20 min, washed three times with PBS, and incubated with DAPI solution for 10 min. Following nuclear staining, cells were washed and mounted on microscope slides using fluorescence mounting medium. All experimental procedures were performed under minimal light conditions to prevent photobleaching. Fluorescence images were acquired using an A1/Ni-E microscope (Nikon, Tokyo, Japan) and analyzed with ImageJ software.

Live imaging of Aβ clearance by BV2 microglial cells was performed under comparable experimental conditions. BV2 microglial cells were cultured on confocal dishes and treated with 200 ng/mL LPS or PBS for 30 min, followed by treatment with 1 μM Entrectinib or 1% DMSO as a vehicle for 23.5 h prior to Aβ exposure. Cells were subsequently exposed to pre-incubated Aβ1-42 immediately before observation under a confocal microscope for 1 h. Live imaging was conducted using a Dragonfly 502w high-speed confocal microscope system equipped with a CO_2_- and temperature-regulated chamber (5% CO_2_, 37 °C). Video recordings and snapshot images were analyzed using Imaris software.

### Y-maze

The effect of Entrectinib on LPS-induced impairments in short-term and spatial memory was evaluated in wild-type mice using the Y-maze test. The maze consisted of three arms measuring 35 × 7 × 15 cm, positioned at 120° angles. Mice were allowed to freely explore the maze for 5 min per session. Spontaneous alternations were recorded using an EthoVision XT video camera (Noldus, Leesburg, VA, USA) and manually quantified. The percentage of alternation was calculated using the following formula:

$${\text{Alternation percentage }}{\mkern 1mu} (\% ) = \left( {{\text{number of alternations}}/{\text{number of alteration trials}}} \right) \times 100\%$$

### Novel object recognition (NOR) test

To evaluate the effects of Entrectinib on long-term and recognition memory, the NOR test was employed. The NOR apparatus consisted of an open-field box measuring 40 × 40× 25 cm. The procedure comprised two phases, training and testing, separated by a 24 h interval. During the training phase, two identical objects were placed in the box, and an individual mouse was allowed to explore for 5 min. During the testing phase, the mouse was reintroduced to the box containing one familiar object and one novel object for an additional 5 min. Object positions during the testing phase were counterbalanced across trials to minimize positional bias. The box and objects were cleaned with 70% ethanol between trials to eliminate residual odor cues. Exploration time was quantified manually by analyzing video recordings of each session. Exploratory behavior was defined as the mouse directing its nose toward an object. Preference for the novel object was calculated using the following formula:

$${\text{Object preference }}{\mkern 1mu} (\% ) = \{ T_{{{\mathrm{Novel}}}} / \, (T_{{{\mathrm{Familiar}}}} + T_{{{\mathrm{Novel}}}} )\} \times 100\%$$

where *T*_*Novel*_ represents the time spent exploring the novel object, and *T*_Familiar_ represents the time spent exploring the familiar object.

### RNA sequencing analysis of mouse primary microglia

Primary microglia isolated from the brains of C57BL/6 J mice were treated with LPS or PBS for 30 min, followed by Entrectinib or DMSO for 23.5 h. Total RNA was subsequently extracted from these microglial cells. RNA integrity was assessed using an Agilent 2100 Bioanalyzer, and all samples exhibited RNA integrity number values above 8. Poly(A) mRNA was isolated from total RNA and fragmented using the Illumina TruSeq Stranded mRNA Library Prep Kit according to the manufacturer’s instructions. Adapter-ligated libraries were sequenced on an Illumina NovaSeq 6000 platform (Macrogen, Korea). mRNA sequencing was performed with three independent biological replicates per condition. Adapter sequences (TruSeq universal and indexed adapters) were removed from raw reads using cutadapt software (version 4.5; https://cutadapt.readthedocs.io/en/stable/). The remaining reads were aligned to the *Mus musculus* reference genome (GRCm39) using STAR software (version 2.7.10) with default parameters (Dobin et al. 2013). After alignment, reads mapped to gene features (GTF file GRCm39.112) were quantified using HTSeq software (Anders et al. 2015).

### Identification of differentially expressed genes (DEGs)

Differential expression analysis was conducted using the Bioconductor package DESeq2 (version 1.38) (Love et al. 2014). DEGs were defined based on adjusted p-values < 0.01 and absolute log2-fold changes > 1 (i.e., twofold change). Gene set enrichment analysis of DEGs was performed using DAVID software (Sherman et al. 2022). Gene Ontology Biological Processes with p-values < 0.05 were considered significantly enriched. Cytoscape was used to reconstruct network models in which nodes were organized according to gene associations and pathway localization within Kyoto Encyclopedia of Genes and Genomes (KEGG) pathways (Kanehisa et al. 2021).

### Statistical analysis

Statistical analyses were performed using GraphPad Prism 9 software (GraphPad Software, San Diego, CA, USA). Comparisons between two groups were conducted using an unpaired two-tailed t-test with Welch’s correction. For multiple-group comparisons, one-way analysis of variance followed by Tukey’s test was applied. Statistical significance was defined as p < 0.05. Data are presented as mean ± SD (*p < 0.05, **p < 0.01, ***p < 0.001, **** p < 0.0001).

## Results

### Entrectinib reduces TRK phosphorylation and proinflammatory factors while enhancing the anti-inflammatory cytokine IL-3 in LPS-treated primary microglia

**Fig. 1:**
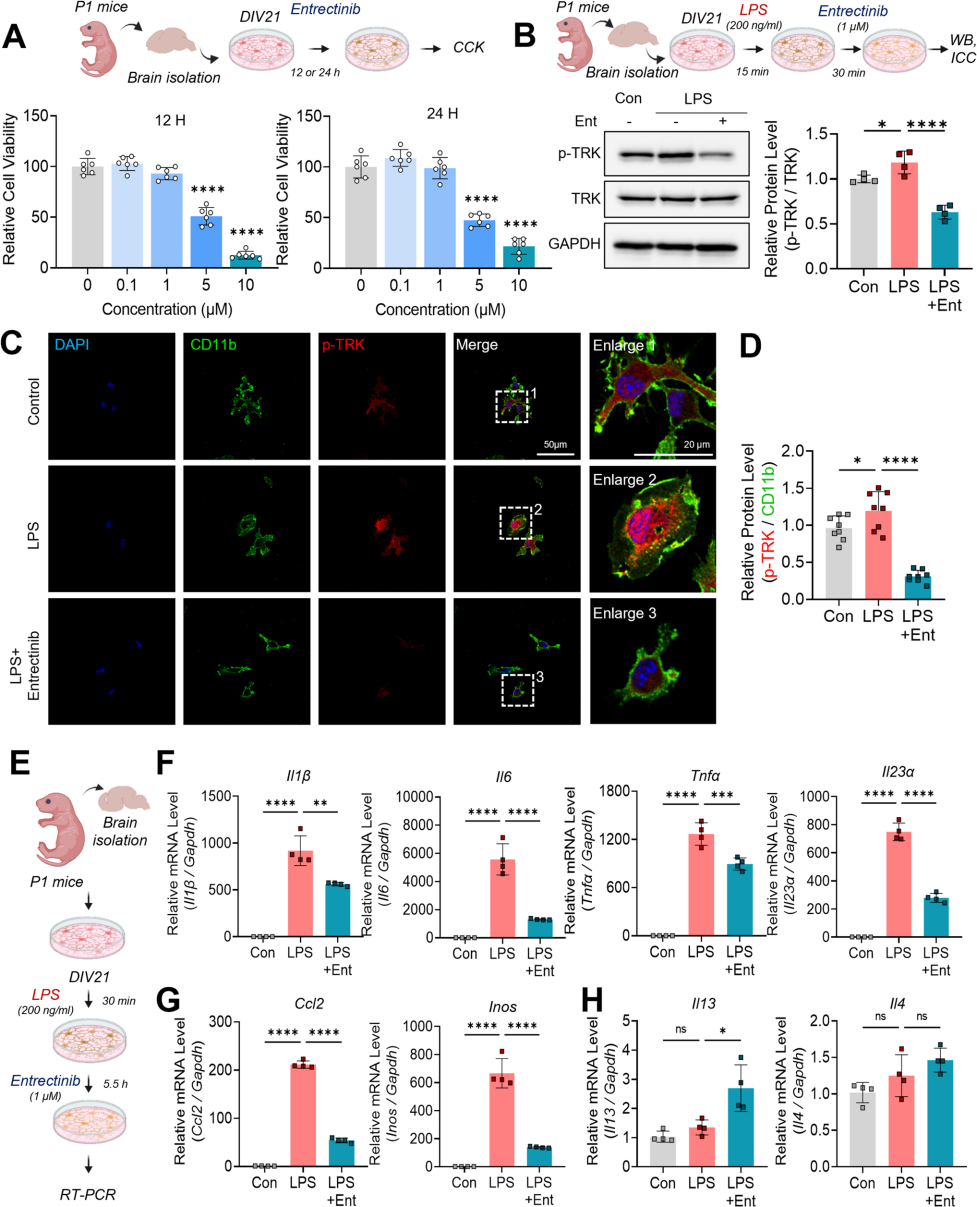
Entrectinib suppresses LPS-induced increases in TRK phosphorylation and proinflammatory factors in primary microglia from mice. **A** Experimental schematic illustrating the CCK-8 assay and evaluation of the cytotoxic effects of Entrectinib in primary microglia. Treatment with 5 and 10 μM Entrectinib reduced cell viability at 12 or 24 h, as assessed by the CCK-8 assay (n = 6/group). **B**, **C** Entrectinib attenuated LPS-induced increases in p-TRK levels in primary microglia (WB: n = 4/group; ICC: n = 8/group). **D** Quantitative analysis demonstrated that Entrectinib reduced LPS-stimulated p-TRK levels in primary microglia. **E** Experimental schematic of LPS and Entrectinib treatments followed by real-time PCR analysis. **F**, **G**. Entrectinib exposure decreased the expression of proinflammatory genes, *Il1β, Il6, Tnfα, Il23α, Ccl2,* and *Inos*, following LPS treatment in primary microglia (n = 4/group). **H** Entrectinib increased anti-inflammatory gene expression, including *Il13* but not *Il14,* in primary microglia (n = 4/group). All values are represented as the mean ± SD. *p < 0.05, **p < 0.01, ***p < 0.001, ****p < 0.0001. *WB* western blotting, *ICC* immunocytochemistry

The role of Entrectinib in neuroinflammation-associated neurodegenerative diseases, such as Alzheimer’s or Parkinson’s diseases, remains unclear. To examine Entrectinib-associated neuroinflammatory responses in microglia, LPS was used to induce microglial activation in vitro. Initial experiments evaluated the cytotoxic effects of Entrectinib on primary microglia by exposing cells to concentrations ranging from 0 to 10 μM for 12 or 24 h. Entrectinib treatment for 12 and 24 h significantly reduced primary microglial viability at 5 μM (Fig. 1A). Based on these findings, a concentration of 1 μM was selected for subsequent experiments, as it did not affect microglial viability.

Entrectinib is known to dominantly antagonize TRKs (Cocco et al. 2018). Based on prior studies, appropriate time points for assessing TRK phosphorylation were selected (Guo et al. 2006; Iyer et al. 2016; Mitre et al. 2022). To determine whether Entrectinib inhibits TRK activation in microglia, primary microglial cultures were treated with 200 ng/mL LPS or PBS for 15 min, followed by exposure to 1 μM Entrectinib or 1% DMSO for 30 min (Fig. 1B). Western blot analysis demonstrated that Entrectinib markedly reduced TRK autophosphorylation in LPS-treated primary microglia (Fig. 1B). Consistent with these findings, immunocytochemical analysis showed a significant reduction in TRK surface phosphorylation following Entrectinib treatment in LPS-stimulated microglia (Fig. 1C and D). Together, these results indicate that Entrectinib modulates microglial activation by antagonizing TRK signaling.

Furthermore, among TRK antagonists, Entrectinib was compared with another clinically relevant TRK inhibitor, Larotrectinib, to evaluate the relative efficacy of TRK antagonism (Dunn 2020). For this comparison, primary microglial cells were treated with LPS or PBS for 15 min, followed by exposure to Larotrectinib (1 or 10 μM) or Entrectinib, as described above. Both Entrectinib and Larotrectinib significantly attenuated LPS-induced TRK phosphorylation. Notably, Entrectinib produced a greater reduction in TRK phosphorylation than either concentration of Larotrectinib (Fig. S1A). These results indicate that Entrectinib exhibits strong potency in inhibiting TRK activation in microglial cells.

Although Entrectinib has been extensively studied in cancer therapy, its effects on inflammatory cytokine regulation in microglia remain largely unexplored. To examine the impact of Entrectinib on microglial inflammatory responses, primary microglial cells were exposed to 200 ng/mL LPS or PBS for 30 min, followed by treatment with 1 μM Entrectinib or 1% DMSO for 5.5 h, based on previously established time points (Kim et al. 2022c). Real-time PCR analysis was performed to assess the expression of proinflammatory cytokine genes (Fig. 1E). Entrectinib significantly attenuated LPS-induced increases in *Il1β, Il6, Tnfα, Il23α*, the chemokine *Ccl2*, and the proinflammatory molecule *Inos* (Fig. 1F and G). In contrast, Entrectinib significantly increased expression of the anti-inflammatory cytokine *Il13, whereas Il4* was unchanged (Fig. 1H). Collectively, these findings demonstrate that Entrectinib suppresses proinflammatory gene expression while selectively enhancing anti-inflammatory signaling in activated microglia.

### Entrectinib reduces LPS-induced phosphorylation of JNK, p38, and AKT in primary microglia

**Fig. 2:**
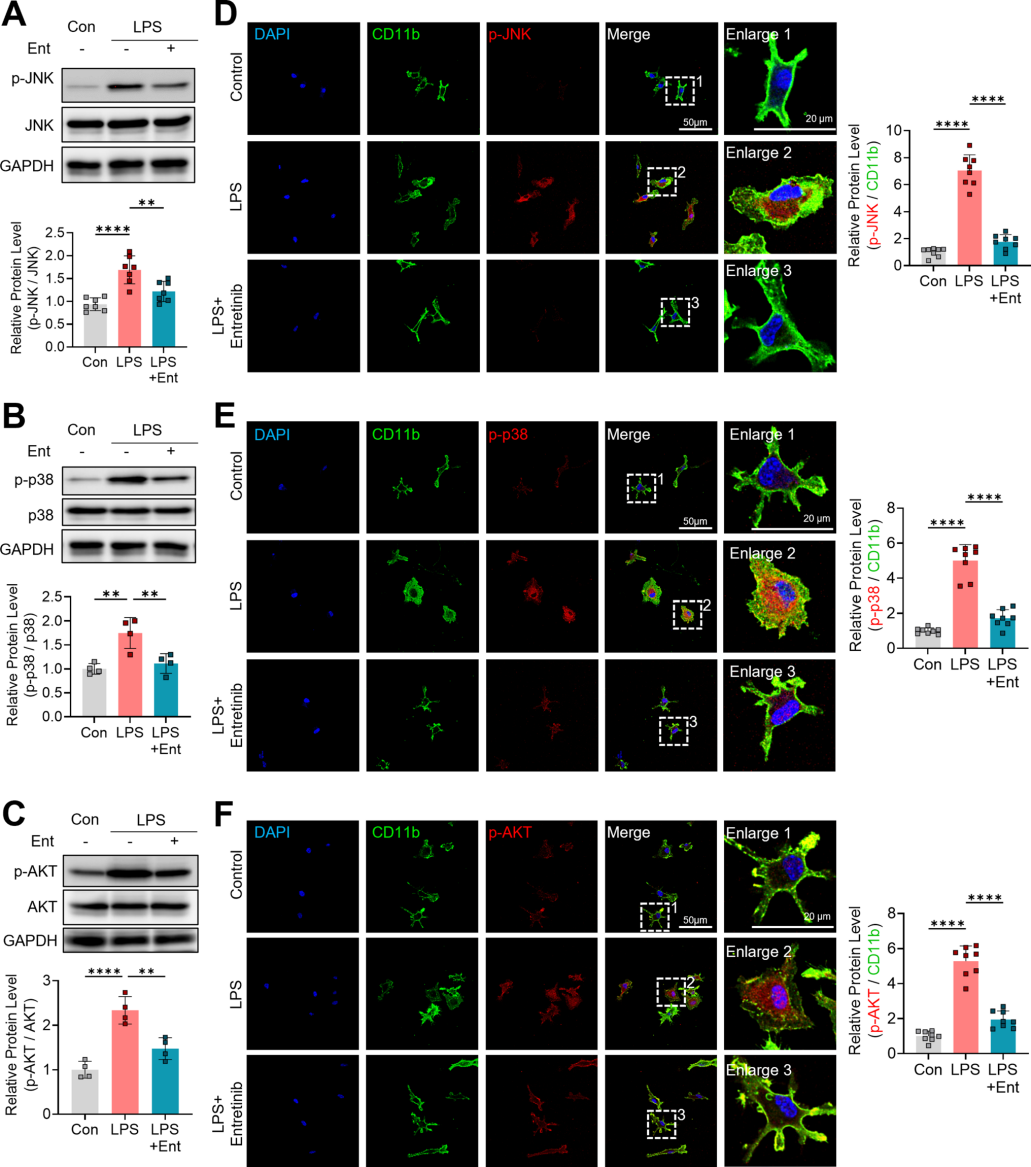
Entrectinib downregulates LPS-stimulated p-JNK, p-p38, and p-AKT protein levels in primary microglia. A-C. Entrectinib reduced LPS-stimulated phosphorylation of JNK, P38, and AKT in primary microglia (JNK, n = 7/group; P38 and AKT, n = 4/group). **D**, **F.** Entrectinib exposure reduced the fluorescence intensity of p-JNK, p-P38, and p-AKT in primary microglia (n = 8/group). All values are presented as the mean ± SD. **p < 0.01, ****p < 0.0001

Multiple signal transduction pathways associated with neuroinflammation have been identified in microglia (Guo et al. 2022; Zhang et al. 2023). In particular, inflammatory signaling in microglia is predominantly mediated through the MyD88-mitogen-activated protein kinase (MAPK) pathways, including JNK, p38, and extracellular signal-regulated kinases (ERK), as well as the PI3K-AKT pathway, which collectively regulate the expression of pro- and anti-inflammatory cytokines and chemokines (Chu et al. 2021; Guo et al. 2024). To investigate the signaling mechanisms underlying Entrectinib-mediated regulation of microglial activation, phosphorylation levels of LPS-induced inflammatory signaling molecules were examined following Entrectinib treatment. Primary microglial cells were treated with 200 ng/mL LPS or PBS for 15 min and subsequently post-treated with 1 μM Entrectinib or 1% DMSO for 30 min (You et al. 2018; Kim et al. 2022a). Cells were then lysed, and protein phosphorylation was analyzed using western blotting. LPS treatment significantly increased phosphorylation of JNK, p38, and AKT, whereas Entrectinib markedly reduced the levels of p-JNK, p-p38, and p-AKT in LPS-stimulated microglia (Fig. 2A and C). These findings were further supported by immunocytochemical analysis, which demonstrated a significant reduction in cytosolic phosphorylation of JNK, p38, and AKT following Entrectinib treatment (Fig. 2D and F). Collectively, these results indicate that Entrectinib attenuates microglial neuroinflammatory signaling by suppressing MAPK and AKT pathway activation.

Next, the effects of Entrectinib on neuroinflammation-related signaling pathways were compared with those of Larotrectinib. As described above, primary microglial cells treated with LPS in the presence of Entrectinib or Larotrectinib were subjected to western blot analysis. LPS-induced increases in p-AKT and p-JNK were significantly reduced by both Entrectinib and Larotrectinib at concentrations of 1 and 10 μM (Fig. S2A, B). Notably, Entrectinib more effectively suppressed AKT phosphorylation than Larotrectinib at equivalent concentrations (Fig. S2). In addition, treatment with 1 μM Entrectinib and 10 μM Larotrectinib significantly reduced LPS-induced p-p38 levels, whereas 1 μM Larotrectinib did not alter p38 phosphorylation (Fig. S2C). These results indicate that Entrectinib more robustly inhibits AKT and p38 signaling pathways in activated microglia.

To further assess whether suppression of downstream inflammatory signaling is mediated through TRK inhibition, TRK siRNA–mediated knockdown experiments were performed in primary microglia. Genetic silencing of TRK significantly reduced LPS-induced phosphorylation of JNK, p38, and AKT compared with cells transfected with scrambled siRNA, supporting the role of TRK as an upstream regulator of these inflammatory signaling pathways (Fig. S3A). Notably, Entrectinib treatment produced a more pronounced reduction in phosphorylation of TRK, JNK, p38, and AKT than TRK siRNA alone, suggesting that Entrectinib-mediated suppression of downstream signaling involves both TRK-dependent and TRK-independent mechanisms (Fig. S3A, B).

### Entrectinib attenuates LPS-induced nuclear translocation of p-NF-κB and p-STAT3 in primary microglia

**Fig. 3:**
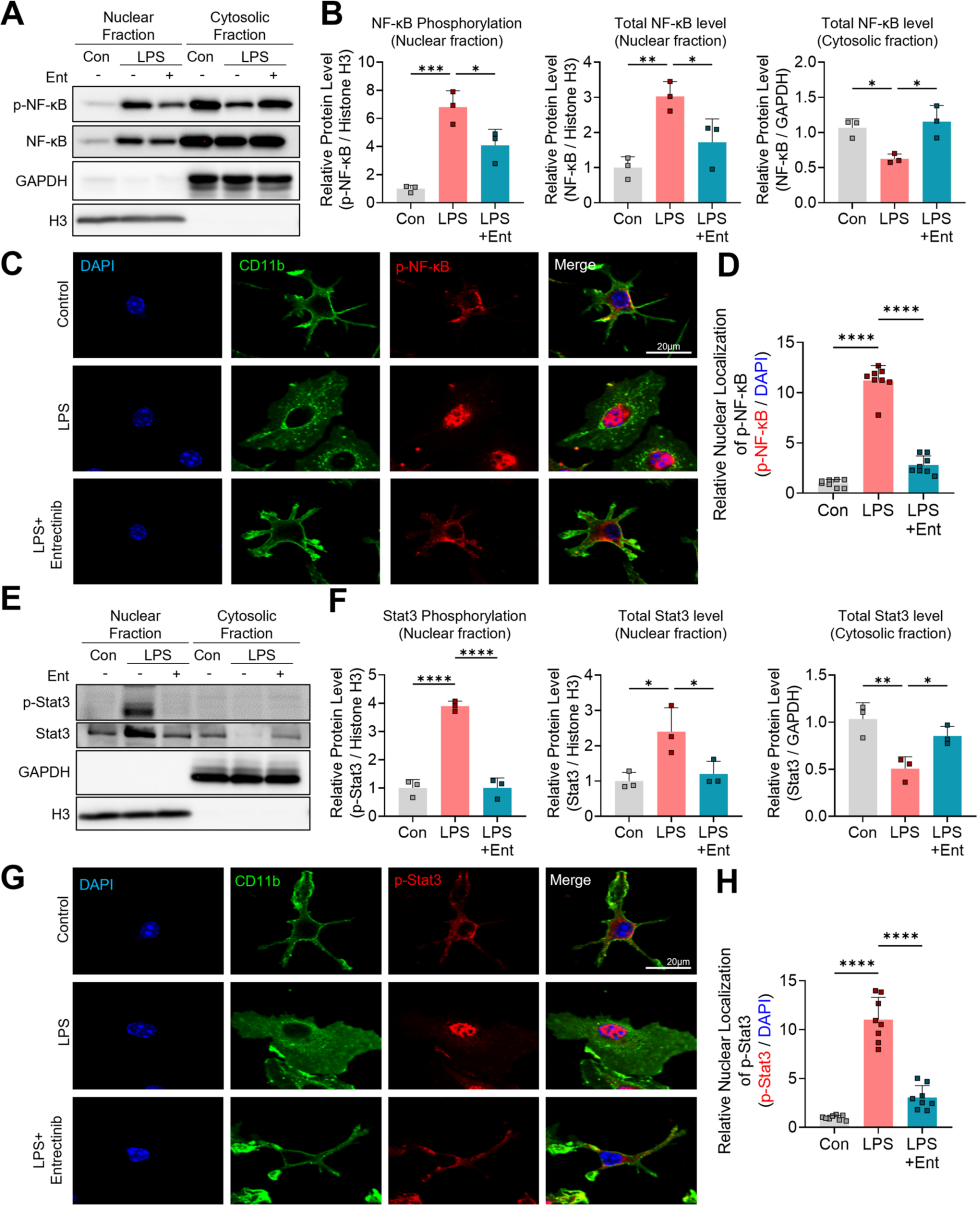
Entrectinib diminishes LPS-evoked nuclear translocation of p-NF-κB and p-STAT3 in primary microglia. **A** and **E** Entrectinib inhibited LPS-induced increases in p-NF-κB and p-STAT3 within the nuclear fraction while augmenting phosphorylation levels in the cytosolic fraction. **B** and **F** Quantitative analysis of panels A and E (n = 3/group). **C** and **G**. Entrectinib reduced nuclear translocation and fluorescence intensity of p-NF-κB and p-STAT3 in primary microglia. **D** and **H** Quantitative analysis of panels **C** and **G** (n = 8/group). All values are presented as the mean ± SD. *p < 0.05, **p < 0.01, ***p < 0.001, ****p < 0.0001

Given that Entrectinib suppresses LPS-induced activation of inflammation-related signaling pathways, including MAPKs and AKT, we next examined the involvement of downstream transcription factors associated with neuroinflammatory responses. Primary microglial cells were exposed to 200 ng/mL LPS or PBS for 30 min, followed by treatment with 1 μM Entrectinib or 1% DMSO for 5.5 h (Kim et al. 2022a). Western blot analysis of cytosolic and nuclear fractions revealed that Entrectinib markedly reduced LPS-induced increases in nuclear p-NF-κB and p-STAT3 levels, while concurrently increasing their cytosolic phosphorylation relative to LPS treatment alone (Fig. 3A, B and E, F). Consistent patterns were observed for total NF-κB and STAT3 proteins in nuclear and cytosolic fractions (Fig. 3A, B and E, F), indicating that LPS promotes nuclear translocation of NF-κB and STAT3, whereas Entrectinib treatment favors their retention in the cytosol. These findings were further supported by immunocytochemical analysis, which demonstrated a significant reduction in nuclear p-NF-κB and p-STAT3 localization following Entrectinib treatment in LPS-stimulated microglia (Fig. 3C, D and G, H). Collectively, these results indicate that Entrectinib modulates microglial neuroinflammatory responses by inhibiting NF-κB and STAT3 nuclear translocation downstream of TRK-associated signaling pathways.

Since Entrectinib regulates transcription factor activity under neuroinflammatory conditions, we next examined whether phosphorylation and nuclear translocation of NF-κB and STAT3 are differentially modulated through inhibition of upstream signaling pathways, including JNK, p38, and AKT. To address this, primary microglial cells were treated with 200 ng/mL LPS or PBS for 30 min and subsequently incubated with 1 μM Entrectinib, 10 μM MK2206 (AKT inhibitor), 20 μM SP600125 (JNK inhibitor), 40 μM SB203580 (p38 inhibitor), or 1% DMSO following LPS or PBS stimulation. Inhibitor concentrations and incubation times were selected based on prior studies (Wang et al. 2010; Cai et al. 2011; Yang et al. 2016; He et al. 2018; Gao et al. 2020). After confirming that each inhibitor selectively reduced phosphorylation of its respective target kinase (p-AKT, p-JNK, or p-p38) (Fig. S4), immunocytochemical analysis was performed to assess transcription factor localization. Nuclear levels of p-NF-κB were significantly reduced by all treatments except SP600125 (Fig. S5A, B). In parallel, nuclear translocation of p-STAT3 was inhibited by all inhibitors examined (Fig. S6A, B). Notably, Entrectinib treatment suppressed nuclear p-STAT3 localization more effectively than SP600125. Collectively, these findings indicate that Entrectinib robustly restricts nuclear localization of NF-κB and STAT3 through coordinated inhibition of upstream kinase-dependent phosphorylation pathways.

### Entrectinib decreases CD16/32 and increases CD206 protein levels in primary microglia

**Fig. 4:**
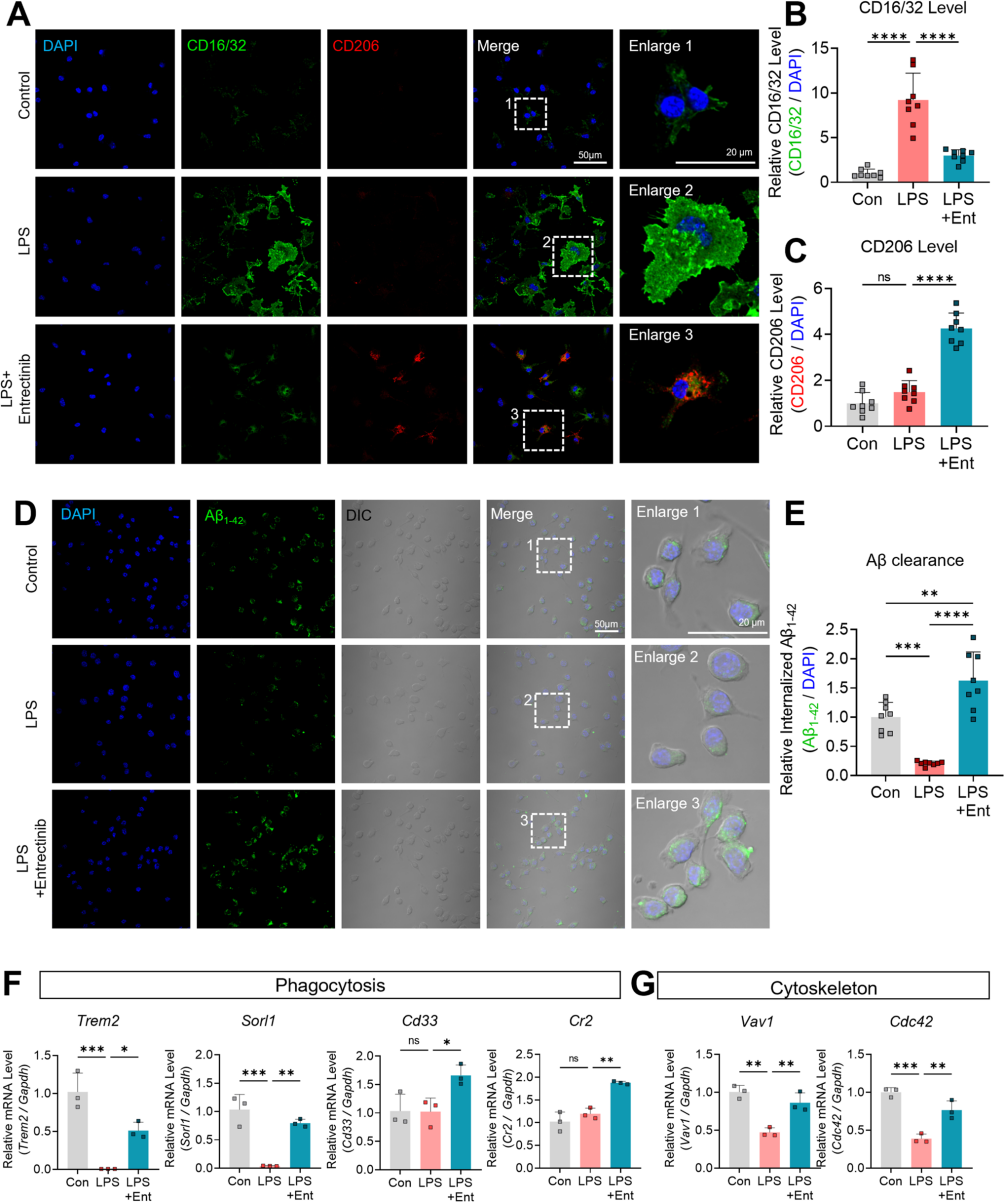
Entrectinib modulates the expression of CD16/32 and CD206 and enhances phagocytic activity in primary microglia. **A** Immunocytochemistry of primary microglia using three markers: DAPI (nuclear marker, blue), CD16/32 (reactive microglial marker, green), and CD206 (anti-inflammatory microglial marker, red). Cells within white dashed rectangles are shown at higher magnification. **B**, **C** Entrectinib treatment decreased expression of the reactive microglial marker CD16/32 and increased expression of the protective microglial marker CD206 in primary microglia, respectively (n = 8/group). **D** Live-imaging analysis of microglial phagocytosis following sequential treatment with LPS, Entrectinib, and Aβ oligomer in BV2 microglial cells. Internalized Aβ1-42 (green) accumulates within the cells. Cells within white dashed rectangles are shown at higher magnification. **E** Quantitative analysis revealed increased internalization of Aβ1-42 following Entrectinib treatment in primary microglia (n = 8/group). **F**, **G** LPS-induced reductions in phagocytosis-related factors and cytoskeletal gene expression were reversed following Entrectinib treatment in primary microglia (n = 3/group). All data are presented as the mean ± SD. *p < 0.05, **p < 0.01, ***p < 0.001, ****p < 0.0001

In this study, Entrectinib attenuated microglial neuroinflammatory signaling by suppressing MAPK- and AKT-associated NF-κB and STAT3 activation. Previous work has demonstrated that microglia adopt distinct phenotypic states that are critical for maintaining inflammation-related homeostasis in the brain. Among these states, phenotypes associated with proinflammatory responses and those linked to resolution and tissue repair are well characterized (Hu et al. 2015). Accordingly, we next examined whether Entrectinib modulates microglial phenotypic markers under LPS-stimulated conditions. Primary microglial cells were treated with 200 ng/mL LPS or PBS for 30 min, followed by exposure to 1 μM Entrectinib or 1% DMSO for 23.5 h. Immunocytochemical analysis was performed using the reactive microglial marker CD16/32 and the anti-inflammatory microglial marker CD206. Entrectinib treatment significantly reduced LPS-induced CD16/32 fluorescence intensity (Fig. 4A and B). In contrast, CD206 fluorescence intensity was significantly increased following Entrectinib treatment compared with LPS alone (Fig. 4A and C). These findings indicate that Entrectinib contributes to modulating changes in microglial markers associated with the function of microglia.

To further compare the effects of Entrectinib with those of Larotrectinib, another TRK antagonist, primary microglial cells were treated with LPS or PBS followed by 1 μM Entrectinib or 1 or 10 μM Larotrectinib, as described above. Immunocytochemical analysis demonstrated that 1 μM Entrectinib and 10 μM Larotrectinib significantly decreased LPS-induced CD16/32 fluorescence intensity. Moreover, treatment with 1 μM Entrectinib or 10 μM Larotrectinib significantly restored CD206 fluorescence intensity that was reduced by LPS stimulation (Fig. S7A–C). Collectively, these results suggest that Entrectinib effectively contributes to the expression of markers in microglia toward an anti-inflammatory state under neuroinflammatory conditions.

### Entrectinib enhances microglial phagocytosis and promotes Aβ clearance

Given that Entrectinib modulates microglia phenotypes through TRK-linked inflammatory signaling pathways, we next examined its effects on microglial phagocytic activity. Initial experiments evaluated whether Entrectinib alters basal microglial phagocytic capacity. For this purpose, BV2 microglial cells were exposed to 250 nM Alexa Fluor 488-conjugated Aβ1-42 oligomers or vehicle for 1 h, followed by live-cell imaging. Representative images demonstrated that Entrectinib treatment significantly increased microglial uptake of Aβ1-42 compared with vehicle-treated controls (Fig. S8A). We then assessed the impact of Entrectinib on microglial phagocytosis under LPS-induced neuroinflammatory conditions. BV2 microglial cells were treated with 200 ng/mL LPS or PBS for 30 min, followed by incubation with 1 μM Entrectinib or 1% DMSO for 23.5 h. During the final hour of treatment, Alexa Fluor 488-conjugated Aβ1-42 oligomers were administered. Live-cell imaging revealed intracellular accumulation of Aβ1-42 within microglial cells during this period. Notably, Entrectinib treatment significantly reversed the LPS-induced reduction in intracellular Aβ1-42 fluorescence intensity, indicating enhanced phagocytic uptake and retention of Aβ1-42 oligomers within BV2 microglia (Fig. 4D and E). Consistent with these observations, temporal fluorescence analysis showed that Entrectinib significantly increased Aβ1-42 fluorescence intensity compared with LPS treatment alone (Fig. S8B). Collectively, these results suggest that Entrectinib restores microglial phagocytic function under inflammatory conditions and promotes Aβ clearance.

To determine whether enhanced microglial phagocytosis is a general effect of TRK inhibition or a property specific to Entrectinib, we compared the effects of Entrectinib and Larotrectinib on Aβ clearance. BV2 microglial cells were treated with LPS or PBS for 30 min, followed by incubation with 1 μM Entrectinib, 1 or 10 μM Larotrectinib, or 1% DMSO for 23.5 h, after which Aβ1-42 oligomers were administered. Quantitative analysis of Alexa Fluor 488 fluorescence revealed a significant increase in intracellular Aβ1-42 signal exclusively in Entrectinib-treated cells compared with all other groups (Fig. S9A and B). These results indicate that Entrectinib uniquely enhances microglial phagocytic uptake of Aβ, distinguishing its effects from those of Larotrectinib.

Given that Entrectinib restored phagocytic activity in LPS-treated BV2 microglia, we next examined whether this functional enhancement is associated with changes in expression of phagocytosis-related receptors and cytoskeletal regulators. Primary microglial cells were exposed to 200 ng/mL LPS or PBS for 30 min, followed by treatment with 1 μM Entrectinib or 1% DMSO for 23.5 h. Real-time PCR analysis demonstrated that Entrectinib significantly increased expression of phagocytosis-associated receptors, including *Trem2, Sorl1, Cd33, Cr2* as well as cytoskeleton-related genes, *Vav1*, and *Cdc42*, compared with LPS treatment alone (Fig. 4F–G). Collectively, these findings indicate that Entrectinib enhances microglial phagocytic capacity by upregulating receptor-mediated recognition pathways and cytoskeletal mechanisms required for efficient engulfment.

### Entrectinib suppresses neuroinflammatory gene programs while enhancing phagocytosis-associated transcriptional signatures

**Fig. 5:**
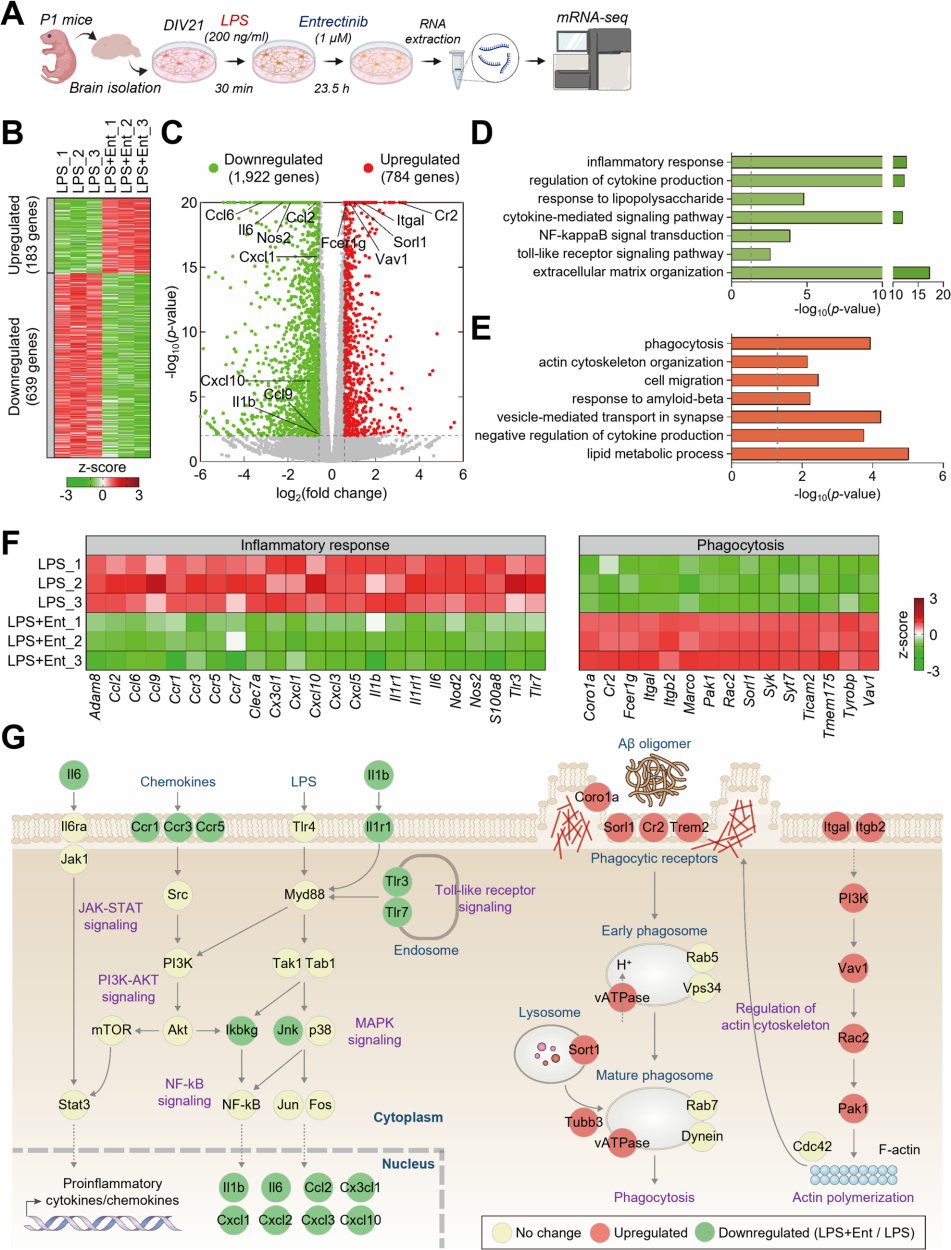
Identification of genes regulated by Entrectinib in LPS-stimulated microglia.** A** Workflow illustrating sample preparation and RNA sequencing. **B** Heatmap showing upregulated and downregulated genes in LPS-stimulated primary microglia treated with Entrectinib compared with DMSO-treated controls (n = 3/group). **C** Volcano plot depicting differentially expressed genes (DEGs) in Entrectinib-treated microglia. The X- and Y-axes represent log2-fold change and-log10(p-value), respectively. Red and green dots indicate upregulated and downregulated genes. Representative proinflammatory and phagocytosis-associated genes are highlighted. **D**, **E**. Gene ontology biological processes (GOBPs) enriched among downregulated **D** and upregulated **E** genes. The x-axis represents − log10(p-value), and the dotted line denotes the p-value cutoff. **F** Heatmap of DEGs associated with inflammatory responses and phagocytosis. The color bar indicates z-score-based normalized expression levels across samples. **G** Network model illustrating interactions among inflammatory and phagocytosis-related signaling pathways. Node colors indicate downregulated (green), upregulated (red), or unchanged (yellow) gene expression in Entrectinib-treated microglia. Nodes are organized based on activation and inhibition relationships derived from the KEGG pathway database. Solid and dotted lines indicate direct and indirect interactions, respectively

To elucidate the molecular mechanisms underlying Entrectinib-mediated suppression of neuroinflammatory responses in primary microglia, RNA sequencing was performed on primary microglial cells treated with LPS followed by Entrectinib or DMSO (Fig. 5A). Comparative transcriptomic analysis identified 2,706 DEGs, including 784 upregulated and 1,922 downregulated genes, in LPS-stimulated microglia treated with Entrectinib relative to DMSO-treated controls (Figs. 5B, C). To characterize the biological processes affected by Entrectinib, functional enrichment analysis of upregulated and downregulated DEGs was performed using DAVID (Kanehisa et al. 2021). Downregulated genes were strongly enriched in inflammation-related processes, including inflammatory response, cytokine production, response to lipopolysaccharide, and cytokine-mediated signaling pathways involving NF-κB and toll-like receptor signaling, as well as extracellular matrix organization (Fig. 5D). In contrast, upregulated genes were predominantly associated with phagocytosis-related processes, including phagocytosis, actin cytoskeleton organization, cell migration, response to amyloid beta, and vesicle-mediated transport (Fig. 5E). Consistent with these pathway-level findings, Entrectinib-treated microglia exhibited reduced expression of proinflammatory genes, including *Ccl2, Ccl6, Ccl9, Cx3cl1, Cxcl1, Cxcl2, Cxcl3, Cxcl10, Il1β, Il6, and Inos* alongside increased expression of genes involved in phagocytosis and immune recognition, such as *Cr2, Fcer1g, Itgal, Itgb2, Sorl1, and Vav1* (Figs. 1F, 4F–G, and 5C). To integrate these transcriptomic changes under LPS-induced pathological conditions, a network model was constructed based on gene–gene interactions annotated in the KEGG database (Kanehisa et al. 2021). The network analysis suggested that Entrectinib suppresses TLR4-dependent signaling and its downstream NF-κB and STAT3 pathways, thereby reducing proinflammatory cytokine and chemokine production, while concurrently enhancing transcriptional programs associated with phagocytosis and actin cytoskeleton organization (Fig. 5G). Collectively, these findings indicate that Entrectinib orchestrates a coordinated transcriptional shift that suppresses neuroinflammatory signaling while promoting microglial phagocytic capacity, consistent with enhanced Aβ clearance under inflammatory conditions.

### Entrectinib attenuates LPS-induced TRK phosphorylation, proinflammatory signaling, and microglial CD16/32 and CD206 marker expression in the hippocampus

**Fig. 6:**
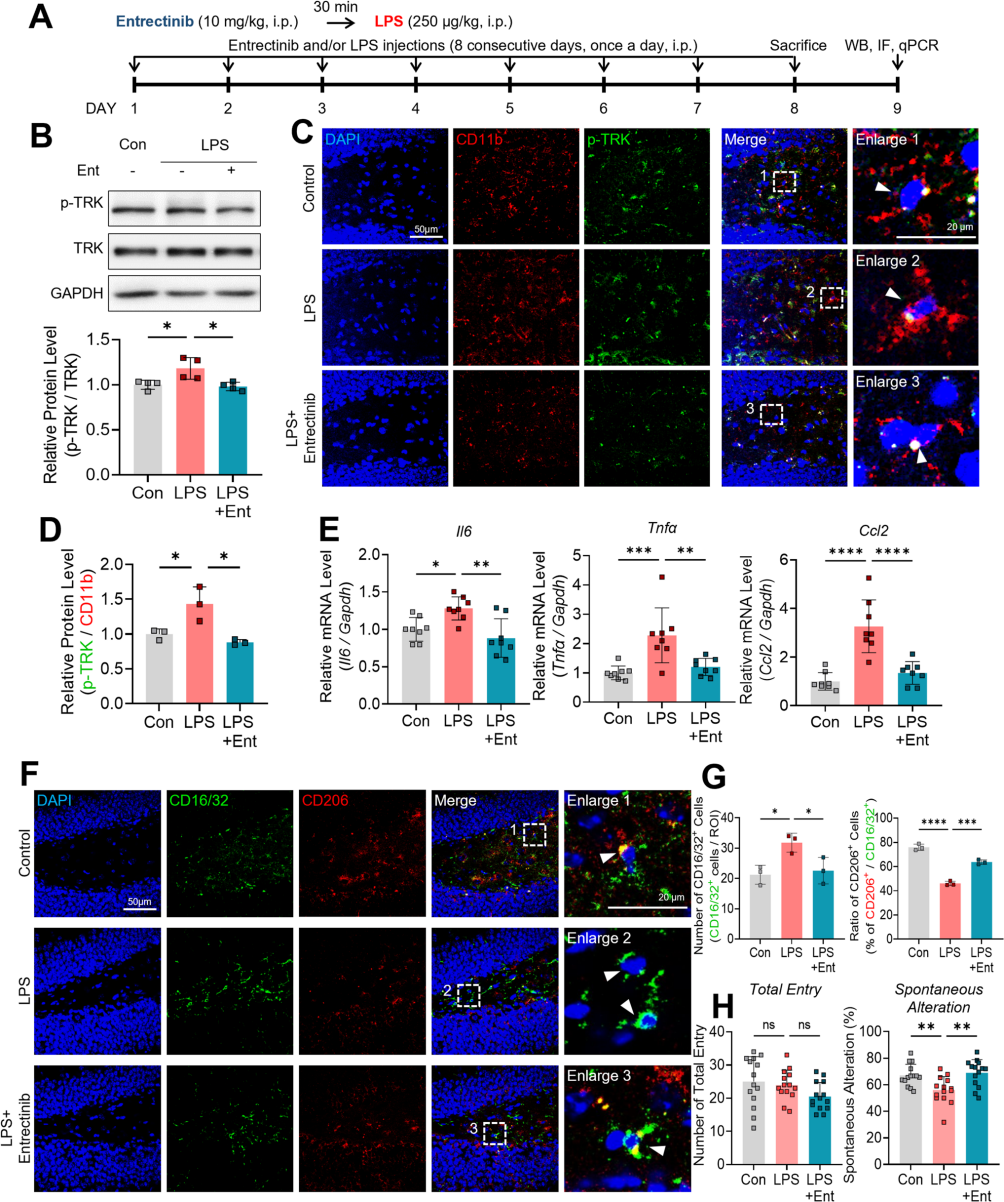
Eight consecutive daily Entrectinib pre-treatments attenuate LPS-induced TRK phosphorylation, suppress neuroinflammatory responses, and improve short-term spatial memory in mice. **A** Schematic diagram illustrating Entrectinib pre-treatment for eight consecutive days in in vivo experiments. **B** Western blot analysis demonstrated that Entrectinib (10 mg/kg, i.p.), administered prior to LPS (250 μg/kg, i.p.) injection, reduced TRK phosphorylation in the hippocampus (n = 4/group). **C** Immunofluorescence staining showing microglial TRK phosphorylation in LPS-injected wild-type mice pre-treated with Entrectinib. Cells within white rectangles are shown at higher magnification. White arrow heads indicate selected nuclear cells. **D** Quantitative analysis of panel C showing that Entrectinib pre-treatment reduced the LPS-induced increase in hippocampal p-TRK levels (n = 3 mice/group, 4 slides/mouse; Control: 10–29 cells/slide; LPS: 31–48 cells/slide; LPS + Ent: 9–35 cells/slide). **E** Entrectinib pre-treatment reduced hippocampal expression of proinflammatory factors Il6, Tnfα, and Ccl2 following LPS challenge (n = 8 mice/group). **F** Immunofluorescence staining showing microglial CD16/32 and CD206 expression in LPS-injected wild-type mice pre-treated with Entrectinib. Cells within white rectangles are shown at higher magnification. White arrow heads indicate selected nuclear cells. **G** Quantitative analysis of panel F demonstrating that Entrectinib exposure decreased CD16/32 and increased CD206 expression in the hippocampus (n = 3 mice/group, 4 slides/mouse; Control: 10–32 cells/slide; LPS: 9–47 cells/slide; LPS + Ent: 9–31 cells/slide). **H** Entrectinib mitigated the LPS-induced reduction in spontaneous alternation performance in the Y-maze, indicating improved short-term spatial memory (n = 14 mice/group). All values are presented as the mean ± SD. *p < 0.05, **p < 0.01, ***p < 0.001, ****p < 0.0001

Given that Entrectinib suppressed LPS-induced neuroinflammatory responses in vitro, we next examined its effects on neuroinflammation in vivo. Eight-week-old male mice received daily intraperitoneal injections of Entrectinib (10 mg/kg) or vehicle (5% DMSO, 40% PEG, and 5% Tween 80 in deionized water), followed 30 min later by intraperitoneal administration of LPS (250 μg/kg) or PBS for 8 consecutive days. On the final day, brains were collected, the hippocampus was isolated, and western blotting, immunofluorescence staining, and real-time PCR analyses were performed (Fig. 6A). Western blot and immunofluorescence analyses demonstrated that Entrectinib significantly reduced LPS-induced TRK phosphorylation in the hippocampus (Fig. 6B–D). Consistent with in vitro findings, LPS-induced upregulation of *Il6*, *Tnfα*, and *Ccl2* expression was significantly attenuated by Entrectinib treatment in hippocampal tissue (Fig. 6E). In addition, phosphorylation of JNK, p38, and AKT was markedly reduced by Entrectinib in the hippocampus (Fig. S10A). Immunofluorescence staining further revealed that LPS robustly increased p-NF-κB and p-STAT3 levels in the hippocampus, whereas these increases were significantly mitigated by Entrectinib treatment (Fig. S10B–E). Together, these data indicate that Entrectinib suppresses hippocampal neuroinflammatory signaling by inhibiting TRK-dependent MAPK/AKT and NF-κB/STAT3 pathways in vivo.

We next examined whether Entrectinib alters microglial phenotype-associated surface markers in the hippocampus. Immunofluorescence analysis revealed that LPS administration significantly increased the number of CD16/32-positive microglia, whereas Entrectinib treatment markedly reduced CD16/32-positive cells in the hippocampus (Fig. 6F–G). Conversely, Entrectinib significantly restored the reduced proportion of CD206-expressing microglia within the total microglial population following LPS exposure (Fig. 6F–G). Collectively, these findings demonstrate that Entrectinib alleviates hippocampal neuroinflammation by suppressing proinflammatory signaling and promoting a change in microglial phenotype toward an anti-inflammatory state in vivo.

### Entrectinib ameliorates LPS-mediated memory impairments in mice

Given that Entrectinib attenuated LPS-induced neuroinflammatory responses in the hippocampus, we next examined whether Entrectinib mitigates neuroinflammation-associated memory impairment in LPS-treated mice. To assess whether suppression of TRK signaling before inflammatory challenge influences cognitive outcomes, Entrectinib was administered as a pre-treatment in this experimental paradigm. Eight-week-old male mice received daily intraperitoneal injections of Entrectinib (10 mg/kg) or vehicle (5% DMSO, 40% PEG, and 5% Tween 80 in deionized water), followed 30 min later by intraperitoneal administration of LPS (250 μg/kg) or PBS for 8 consecutive days. Behavioral assessments, including the Y-maze and NOR tests, were conducted on days 7–8 (Fig. S10F). Repeated LPS administration resulted in transient body weight loss in both the LPS-treated and Entrectinib plus LPS-treated groups; however, by day 7, body weight and locomotor activity returned to baseline levels (Fig. S10G). Y-maze testing performed on day 7 revealed that LPS-induced reductions in spontaneous alternation were significantly ameliorated by Entrectinib treatment compared with the LPS-only group (Fig. 6H). In contrast, Entrectinib pre-treatment did not significantly reverse the LPS-induced reduction in novel object preference during the NOR test (Fig. S10H).

To determine whether prolonged pre-treatment with Entrectinib exerts broader effects on cognitive function, mice were exposed to Entrectinib and LPS over an extended period. Eight-week-old male mice received daily intraperitoneal injections of Entrectinib (10 mg/kg) or vehicle, followed 30 min later by administration of LPS (250 μg/kg) or PBS for 16 consecutive days. Behavioral assessments, including the Y-maze and NOR tests, were conducted on days 15–16 (Fig. S11A). Consistent with shorter-duration treatment, Entrectinib significantly increased spontaneous alternation percentages in the Y-maze compared with LPS-treated mice, indicating improved short-term spatial working memory (Fig. S11C). Notably, prolonged Entrectinib treatment also significantly increased novel object preference in the NOR test relative to LPS treatment alone, reflecting restoration of recognition memory (Fig. S11D). In parallel, Entrectinib exposure markedly reduced phosphorylation of TRK and proinflammatory signaling molecules in the hippocampus following LPS administration (Fig. S11E–F). These findings indicate that sustained Entrectinib pre-treatment more effectively counteracts neuroinflammation-associated deficits in both short- and long-term memory than shorter treatment durations.

**Fig. 7:**
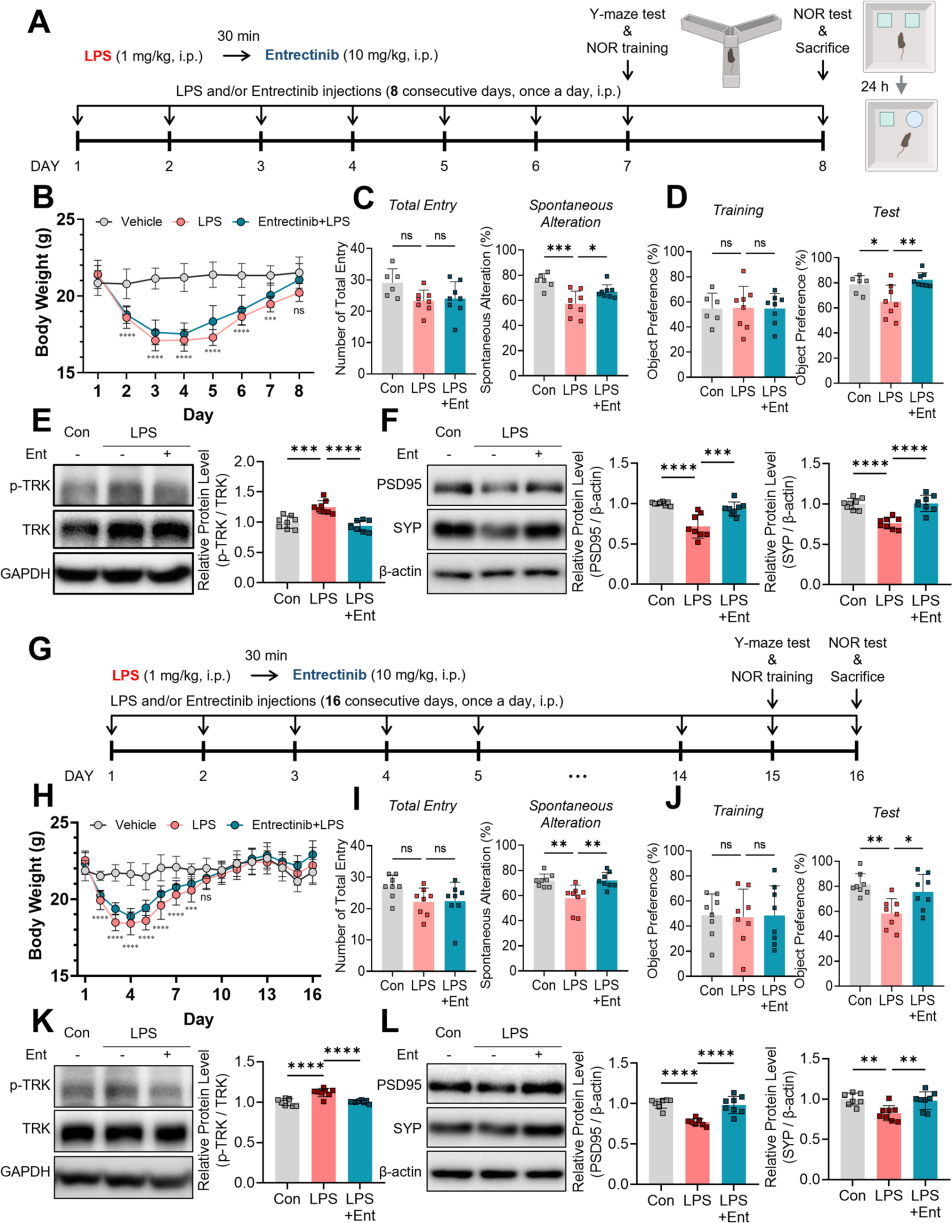
Short- and long-term Entrectinib post-treatment restores LPS-evoked memory impairment in mice. **A** and **G** Schematic diagrams illustrating Entrectinib post-treatment for eight or sixteen consecutive days in in vivo experiments. **B** and **H**. Body weight changes during the LPS and Entrectinib injection period over eight or sixteen days (n = 8–9 mice/group). **C** and **I** Entrectinib post-treatment mitigated the LPS-induced reduction in spontaneous alternation performance, while total arm entries remained unchanged in the Y-maze (n = 6–8 mice/group). **D** and **J** Repeated Entrectinib administration over 8 or 16 days increased the percentage of object preference in the NOR test following LPS treatment (n = 6–8 mice/group). **E** and **K** Short- and long-term Entrectinib post-treatment reduced LPS-evoked p-TRK protein levels in the hippocampus (n = 8 mice/group). **F** and **L**. Entrectinib exposure restored LPS-induced reductions in the presynaptic marker synaptophysin (SYP) and the postsynaptic marker PSD95 in the hippocampus (n = 8 mice/group). All values are presented as the mean ± SD. *p < 0.05, **p < 0.01, ***p < 0.001, ****p < 0.0001

Since pre-administration of Entrectinib restored LPS-induced memory impairments, we next examined whether Entrectinib also exerts beneficial effects when administered after the onset of neuroinflammation. This post-treatment paradigm evaluated the therapeutic potential of Entrectinib under established inflammatory conditions. In this experiment, a higher dose of LPS (1 mg/kg) was used to induce more robust memory impairment. Eight-week-old male mice were injected intraperitoneally with LPS (1 mg/kg) or PBS, followed 30 min later by daily administration of Entrectinib (10 mg/kg) or PBS for either 8 or 16 days. Behavioral assessments using the Y-maze and NOR tests were conducted on days 7–8 or 15–16 (Fig. 7A and G). All mice regained body weight by the time of behavioral testing (Fig. 7B and H). Post-treatment with Entrectinib for both 8 and 16 days significantly increased spontaneous alternation and object preference in the Y-maze and NOR tests, respectively (Fig. 7C and D, I and J). Consistent with pre-treatment findings, Entrectinib post-treatment markedly reduced LPS-induced TRK phosphorylation and proinflammatory factors in the hippocampus (Figs. 7E, K, and S12A–B). These results demonstrate that Entrectinib remains effective when administered after neuroinflammatory initiation.

Memory impairment is associated with alterations in hippocampal dendritic spine formation and synaptic plasticity in the mouse brain (von Bohlen und Halbach et al. 2006; Dorostkar et al. 2015; Frank et al. 2018; Goto 2022). To investigate synaptic changes associated with Entrectinib treatment, western blotting was performed using hippocampal tissue collected from mice after behavioral testing. The presynaptic marker synaptophysin (SYP) and the postsynaptic marker postsynaptic density protein 95 (PSD95) were examined (Glantz et al. 2007; Yuki et al. 2014; Zeng et al. 2015; Wang et al. 2016). Both PSD95 and SYP levels, which were reduced following LPS exposure, were significantly restored after 8 and 16 d of Entrectinib administration (Fig. 7F and L). These findings indicate that both pre- and post-treatment with Entrectinib are associated with recovery of synaptic protein expression, which may underlie the observed behavioral improvements.

## Discussion

**Fig. 8:**
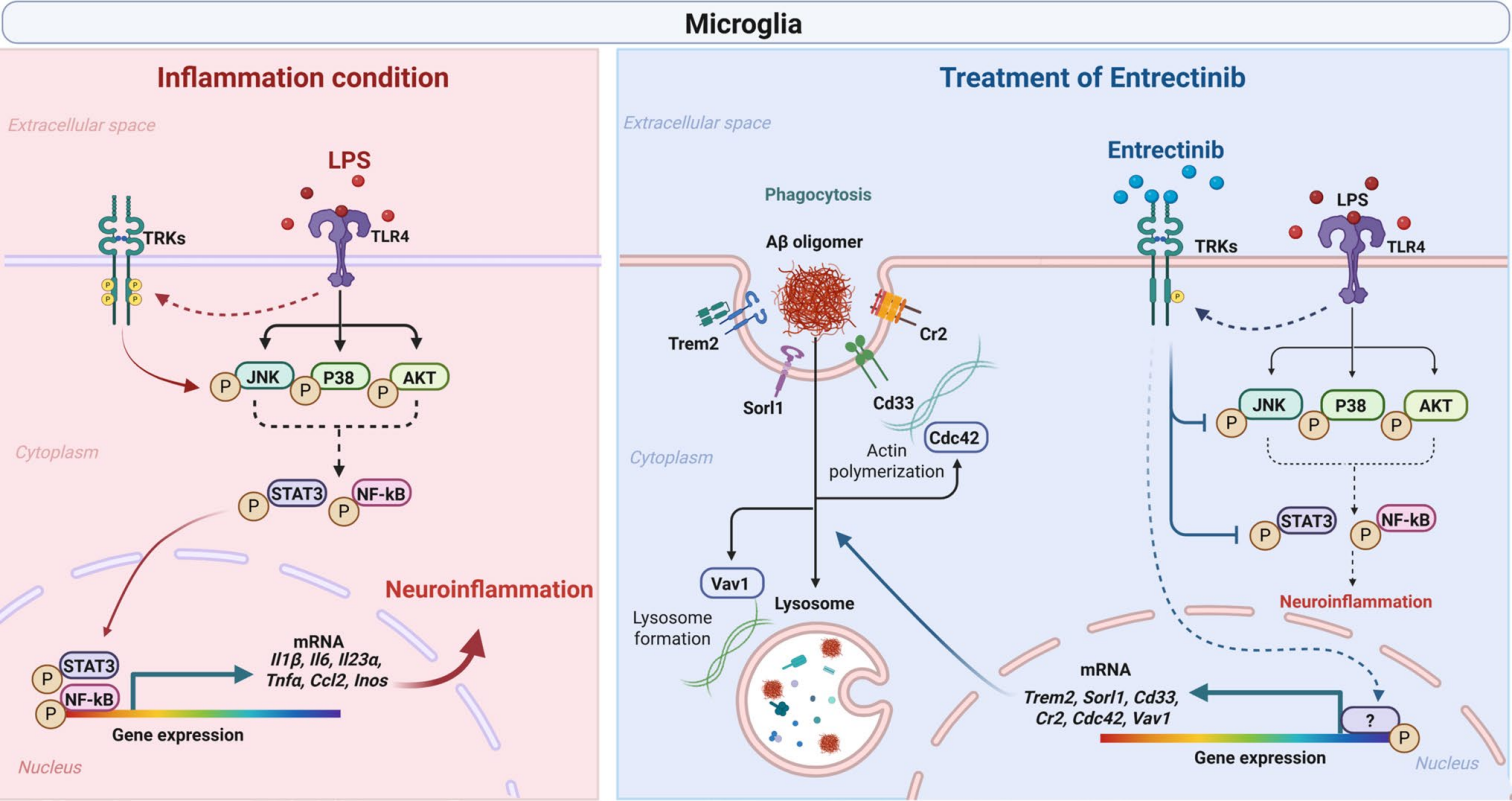
Proposed mechanism underlying Entrectinib-mediated modulation of LPS-induced neuroinflammatory responses in microglia. Under LPS-induced neuroinflammatory conditions, LPS binds to TLR4 and activates the JNK, P38, and AKT signaling pathways. Activation of these pathways promotes phosphorylation of cytosolic NF-κB and/or STAT3, resulting in their translocation into the nucleus. Nuclear NF-κB and STAT3 subsequently drive the transcription of neuroinflammation-associated genes, including *Il1β, Il6, Il23α, Tnfα, Ccl2,* and *Inos,* thereby promoting microglia-mediated neuroinflammatory responses (left panel). In contrast, Entrectinib attenuates LPS-induced inflammatory signaling in microglial cells by inhibiting phosphorylation of JNK, P38, and AKT, which prevents NF-κB and STAT3 nuclear translocation. Therefore, Entrectinib promotes increased expression of phagocytic receptors and cytoskeletal remodeling factors, including actin polymerization regulators, Cdc42, lysosome-associated factors, and Vav1. These coordinated molecular changes support enhanced microglial functional responses (right panel). Collectively, Entrectinib mitigates LPS-induced gliosis in both in vitro and in vivo settings, highlighting its potential to modulate neuroinflammation-associated pathological processes

In this study, we demonstrated that Entrectinib reduces LPS-induced TRK phosphorylation and inflammatory factors by suppressing neuroinflammatory signaling pathways involving p-JNK, p-p38, and p-AKT, which regulate the nuclear transcription factors NF-κB and STAT3 in primary microglia. Entrectinib also modulates LPS-stimulated microglial phenotype markers and enhances phagocytic activity by upregulating genes associated with phagocytosis-related receptors and cytoskeletal remodeling in microglia. In addition, Entrectinib significantly inhibited LPS-induced increases in proinflammatory factors and CD16/32 protein levels, while restoring CD206 expression in hippocampal microglia, consistent with observations in primary microglia. Notably, Entrectinib alleviated LPS-induced impairments in both short- and long-term memory in vivo. Collectively, the ability of Entrectinib to modulate LPS-induced microgliosis in vitro and in vivo, and to ameliorate neuroinflammation-associated behavioral deficits, underscores its potential as a therapeutic agent for neuroinflammation-related brain disorders (Fig. 8).

Entrectinib is a potent multi-kinase inhibitor that targets tyrosine residues and modulates TRK activity in tumor cells (Kita et al. 2017; Suzuki et al. 2022; Chen et al. 2024). TRKs play a critical role in regulating synaptic strength and neuronal plasticity, thereby influencing neuronal signaling and function (Longo and Massa 2013). Beyond the nervous system, TRKs have also been implicated in the regulation of inflammatory responses in immune cells, including macrophages (di Mola et al. 2000; Ley et al. 2013). Tumor-associated macrophages have been shown to increase IL-10, IL-6, and TNF-α production through Trk-A activation (Ley et al. 2013). NGF and Trk-A expression levels are markedly elevated in affected tissues, including regions surrounding enteric glial cells (di Mola et al. 2000). Consistent with these observations, TRK inhibitors such as LOXO-101 and crizotinib significantly reduce inflammatory markers, including COX-2, in colon cancer models (Sohn et al. 2021). Despite this evidence, the effects of Entrectinib on TRK-associated neuroinflammatory pathways in microglia and its actions within the CNS have remained unexplored. Our findings demonstrate that Entrectinib inhibits TRK phosphorylation and proinflammatory factor expression while increasing anti-inflammatory cytokines in primary microglia and the hippocampus of mouse brains (Figs. 1 and 6).

LPS is a well-established pathogen that activates toll-like receptor 4 (TLR4), thereby initiating inflammatory responses in the CNS. Numerous studies have shown that LPS stimulates proinflammatory signaling through TLR4 in both BV2 and primary microglia (Kacimi et al. 2011; Kim et al. 2022a, 2022b, 2022c). For example, treatment with 200 ng/mL LPS markedly increases phosphorylation of p38 and AKT, as well as the transcription factors p-NF-κB, and p-STAT3, in BV2 and primary microglia (Kim et al. 2022c). In addition, exposure to 10 μg/mL LPS induces JNK and p38 phosphorylation in BV2 microglia (Kacimi et al., 2011). However, the effects of Entrectinib on LPS-induced inflammatory signaling in microglia and in the hippocampus, a brain region critical for memory integration, remain unclear. In this study, we found that Entrectinib inhibited LPS-induced increases in p-JNK, p-p38, and p-AKT signaling, as well as p-NF-κB and p-STAT3 activation, in primary microglia and the hippocampus of mice (Figs. 2, 3, and Fig. S10). These findings indicate that Entrectinib attenuates CNS neuroinflammatory responses by suppressing JNK/p38/AKT and NF-κB/STAT3 signaling pathways in both LPS-treated microglia and LPS-injected mouse brains.

Importantly, the concordance between pharmacological inhibition by Entrectinib and genetic suppression of TRK signaling supports a model in which TRK acts as an upstream regulator of JNK, p38, and AKT activation in LPS-stimulated microglia. TRK knockdown using siRNA attenuated phosphorylation of these downstream inflammatory kinases, providing direct causal evidence for TRK-dependent regulation of the JNK/p38/AKT axis (Fig. S3). In contrast, Entrectinib produced a more pronounced reduction in TRK phosphorylation and downstream signaling, suggesting that its anti-inflammatory effects involve both TRK-dependent mechanisms and additional kinase targets. Entrectinib may therefore influence LPS-induced neuroinflammatory factor production by targeting other receptor tyrosine kinases and signaling molecules, including proto-oncogene tyrosine-protein kinase ROS1, anaplastic lymphoma kinase, Janus kinase/ERKs, or mammalian target of rapamycin (mTOR), which are implicated in inflammation-related signaling pathways. Consistent with this possibility, Entrectinib was recently shown to bind the arginine 121 residue of the mitotic serine/threonine kinase NEK7, thereby suppressing NLRP3 inflammasome formation and regulating systemic inflammation in a mouse model of type 2 diabetes (Jin et al. 2023). Accordingly, further studies are required to delineate the relative contributions of TRK-dependent and off-target mechanisms to the anti-inflammatory actions of Entrectinib in LPS-associated signaling contexts.

Triple-immunofluorescence staining revealed that markers of reactive microgliosis were downregulated, whereas markers of protective microglia were upregulated following Entrectinib treatment in both primary microglia and hippocampal microglia (Figs. 4 and 6). In addition, microglial phagocytic activity and the expression of phagocytosis-associated membrane receptors and cytoskeletal components were significantly increased after Entrectinib treatment in microglial cultures (Fig. 4). Notably, Entrectinib significantly enhanced the production of the anti-inflammatory cytokine *Il13* in primary microglia (Fig. 1). These findings are indicative of a transition from a reactive to a resting or protective microglial state, potentially mediated by Il13, which is known to promote anti-inflammatory microglial phenotypes. This interpretation is supported by prior studies showing that reactive microglia shift toward a protective phenotype through polarization mechanisms following exposure to *Il4* and *Il13* in macrophages (Scott et al. 2023). In addition, treatment with IL-4 and IL-10 has been reported to upregulate protective microglial markers, including CD206 and Arg-1, and to enhance phagocytic capacity via time-dependent induction of Trem2 expression (Yi et al. 2020). Taken together with existing literature, our findings suggest that Entrectinib regulates microglial phenotypic markers while enhancing phagocytosis- and cytoskeleton-related gene expression. Recent studies have identified multiple microglial states, including stage 1 and stage 2 disease-associated microglia, lipid droplet–accumulating microglia (LDAM), and the microglia neurodegenerative phenotype (MGnD) (Keren-Shaul et al. 2017; Marschallinger et al. 2020; Wei and Li 2022). Therefore, further studies using expanded marker panels will be required to more precisely define the microglial states modulated by Entrectinib.

A link between neuroinflammation and memory impairment has been proposed, as proinflammatory molecules such as IL-1 and IL-1β are elevated in the brain following LPS injection and in Alzheimer’s disease mouse models (Oitzl et al. 1993; Barrientos et al. 2002; Hein et al. 2010). Both peripheral and intracerebral administration of proinflammatory cytokines, including IL-1β, impair hippocampus-dependent spatial memory in mice (Oitzl et al. 1993; Barrientos et al. 2002; Hein et al. 2010). Consistently, chronic low-dose LPS administration (250 µg/mL, 7 consecutive days, i.p.) significantly reduces short-term spatial memory in mice (Kim et al. 2022c). Several receptor tyrosine kinase inhibitors have been reported to rescue LPS-induced microgliosis and memory impairment in vivo (Kim et al. 2022c; Lee et al. 2022). For example, the SUR1 inhibitor gliquidone significantly reduced LPS-induced increases in Iba-1-positive microglia in the hippocampus following acute LPS administration (10 mg/mL, i.p.) (Kim et al. 2021). Similarly, the EGFR inhibitor varlitinib decreased LPS-stimulated Iba-1-positive cell counts and IL-1β levels in the hippocampus (Kim et al. 2022b). Additionally, the multi-tyrosine kinase inhibitor nilotinib significantly restored short-term spatial memory after 2 weeks of chronic LPS exposure (Kim et al. 2022c). Based on these findings, we hypothesized that Entrectinib could alleviate LPS-induced cognitive deficits by suppressing microgliosis. In the present study, post-treatment with Entrectinib (10 mg/kg, i.p., daily for 8 or 16 d) significantly improved LPS-induced reductions in spontaneous alternation and novel object preference (Fig. 7), although the behavioral effects were modest. Entrectinib treatment was also associated with increased hippocampal levels of the presynaptic marker SYP and the postsynaptic marker PSD95 (Fig. 7), suggesting partial restoration of synaptic integrity. Collectively, these findings indicate that Entrectinib partially ameliorates LPS-induced memory impairment, likely through attenuation of neuroinflammation and modulation of synaptic markers rather than complete recovery of cognitive function in vivo.

This study highlights the therapeutic potential of targeting TRK signaling with Entrectinib in diseases associated with neuroinflammation. Clinical evidence indicates that Entrectinib is generally safe and well tolerated, with no cumulative toxicity reported in the majority of patients with NSCLC (Jiang et al. 2022). In addition, treatment with Entrectinib markedly reduced pulmonary tumor burden in a case of inflammatory myofibroblastic tumor, and prolonged administration was associated with normalization of systemic inflammatory markers (Ambati et al. 2018). Consistent with these observations, our findings demonstrate that Entrectinib significantly modulates neuroinflammatory responses in an LPS-induced mouse model, supporting its potential as a therapeutic candidate for neuroinflammation-related neurodegenerative conditions, including Alzheimer’s and Parkinson’s disease.

An important limitation of this study is that the LPS-induced neuroinflammation model used in vitro primarily reflects an acute or short-term inflammatory response triggered by brief LPS exposure. In contrast, chronic neuroinflammatory disorders, including Alzheimer’s and Parkinson’s diseases, are characterized by persistent and sustained inflammatory processes (Tansey et al. 2022; Heneka et al. 2025). Although LPS-based models are widely used and valuable for dissecting acute or subacute inflammatory signaling pathways, extrapolation of these findings to chronic disease pathology remains challenging. In addition, while our data demonstrate that Entrectinib suppresses TRK phosphorylation and downstream inflammatory signaling pathways, including JNK, p38, and AKT, mechanistic specificity should be interpreted with caution. TRK knockdown using siRNA attenuated activation of JNK, p38, and AKT, supporting a functional role for TRK in regulating these inflammation-associated pathways. However, quantitative analyses showed that Entrectinib reduced TRK phosphorylation more robustly than TRK siRNA alone, raising the possibility that additional kinase targets contribute to its anti-inflammatory effects. Thus, although our findings establish TRK as an upstream regulator of JNK, p38, and AKT signaling during microglial neuroinflammation, the involvement of non-TRK mechanisms cannot be excluded. Future studies employing genetic rescue strategies, kinase-dead TRK mutants, or more selective TRK inhibitors, together with chronic inflammation models and longitudinal analysis of microglial dynamics, will be required to clarify the relative contributions of TRK-dependent and off-target mechanisms to the therapeutic actions of Entrectinib.

In summary, Entrectinib reduces proinflammatory factors while increasing anti-inflammatory cytokine production and enhancing the expression of phagocytosis-related membrane receptors and cytoskeletal genes. These effects are mediated through suppression of LPS-induced JNK, p38, AKT/NF-κB, and STAT3 signaling pathways both in vitro and in vivo. While LPS activates TLR4 signaling and engages TRK-dependent mechanisms, Entrectinib restores microglial function by antagonizing TRK signaling, thereby improving LPS-induced memory impairment. Collectively, these findings indicate that TRK blockade by Entrectinib attenuates microgliosis and represents a potential therapeutic strategy for neuroinflammation-related disorders.

## Supplementary Information

Below is the link to the electronic supplementary material.

Supplementary file1 (DOCX 4747 KB)

Supplementary file2 (MP4 70248 KB)

Supplementary file3 (MP4 79683 KB)

Supplementary file4 (MP4 60940 KB)

Supplementary file5 (XLSX 289 KB)

## Data Availability

All data generated and analyzed during this study are included in this article and its supplementary information files.
