# Supplementary material for: Entrectinib attenuates LPS-induced neuroinflammation by inhibiting JNK, p38, and AKT pathways and ameliorates cognitive impairment
Source: Arch Pharm Res. 2026 Apr 5;49(3):393–415. doi: 10.1007/s12272-026-01608-x (PMC13076427; doi:10.1007/s12272-026-01608-x)
Supplement: Supplementary file 1 — Supplementary file1 (DOCX 4747 KB) [file 12272_2026_1608_MOESM1_ESM.docx]

**Supplementary Information**

**Entrectinib attenuates LPS-induced neuroinflammation by inhibiting JNK, p38, and AKT pathways and ameliorates cognitive impairment**

Hanwoong Woo^1+^, Sung Wook Kim^2+^, Sohee Kim^3^, Sehyun Chae^1,4^* and Jieun Kim^1,3,5^*

^1^ Multidimensional Genomics Research Center, Kangwon National University, Chuncheon 24341, Republic of Korea,

^2^Department of Neurovascular Unit Research Group, Korea Brain Research Institute (KBRI), 61, Cheomdan-ro, Dong-gu, Daegu, 41062, Republic of Korea,

^3^Department of Bio-Health Convergence, Kangwon National University, Chuncheon 24341, Republic of Korea

^4^Division of Chemical Engineering and Bioengineering, College of Art Culture and Engineering, Kangwon National University, Chuncheon, 24341, Republic of Korea,

^5^Department of Bio-Health Technology, College of Biomedical Science, Kangwon National University, Chuncheon, 24341, Republic of Korea,

+ These authors contributed equally to this work.

*Corresponding author:

Sehyun Chae, Ph.D.
Division of Chemical Engineering and Bioengineering,
College of Art, Culture and Engineering,
Kangwon National University,
Chuncheon 24341, Republic of Korea
Telephone: +82-33-250-6274
E-mail: [shchae@kangwon.ac.kr](mailto:shchae@kangwon.ac.kr)

Jieun Kim, Ph.D.
Department of Bio-Health Technology,
College of Biomedical Science,
Kangwon National University,
Chuncheon 24341, Republic of Korea
Telephone: +82-33-250-6479
Fax: +82-33-250-6470

E-mail: [jieunkim@kangwon.ac.kr](mailto:jieunkim@kangwon.ac.kr)


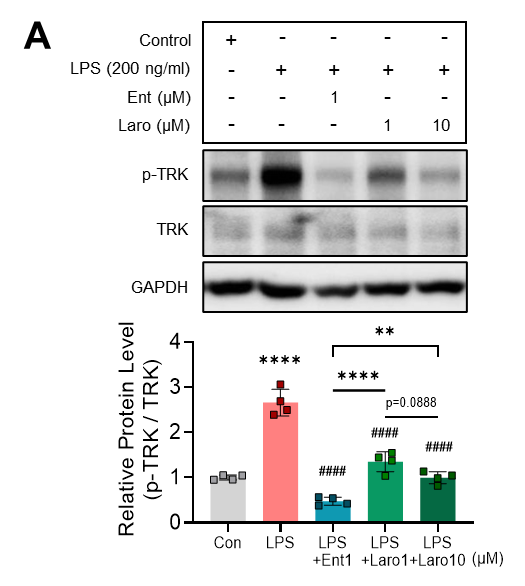


**Supplementary Figure 1. Entrectinib reduces LPS-induced TRK phosphorylation more effectively than Larotrectinib.** **A.** Effects of Entrectinib or Larotrectinib on TRK phosphorylation in LPS-stimulated primary microglial cells from mice, assessed using western blotting. Entrectinib produced a greater reduction in TRK phosphorylation compared with both tested doses of Larotrectinib (n=4/group). All values are presented as the mean ± SD. ****p < 0.0001 vs. control group; ####p < 0.0001 vs. LPS group; comparisons indicated above connecting lines: **p < 0.01, ****p < 0.0001.


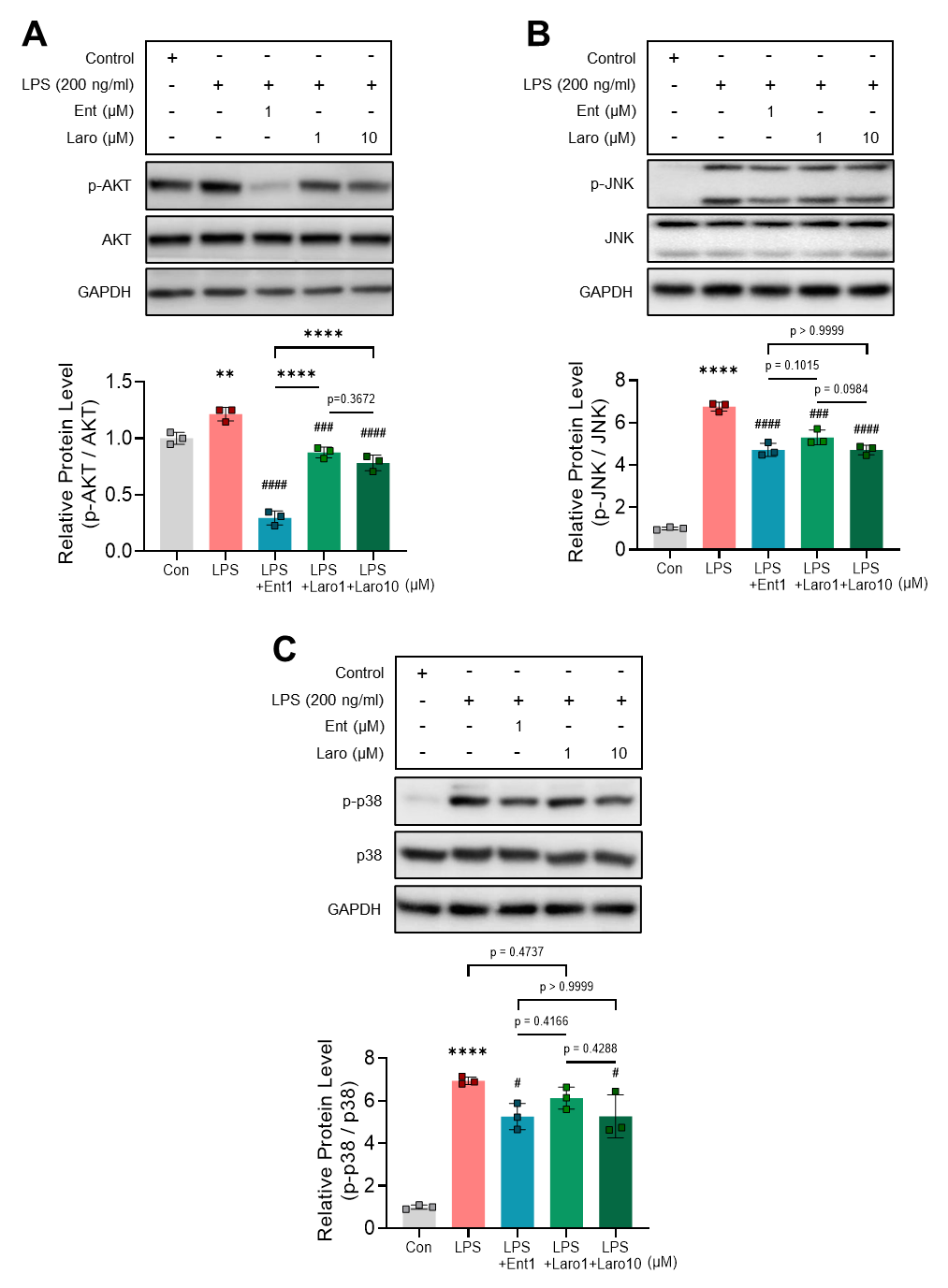


**Supplementary Figure 2. Entrectinib suppresses LPS-stimulated p-AKT, p-JNK, and p-p38 protein levels more effectively than Larotrectinib.** **A-B.** Effects of Entrectinib or Larotrectinib on AKT and JNK phosphorylation in LPS-stimulated primary microglial cells from mice, assessed using western blotting. Entrectinib and both tested doses of Larotrectinib reduced LPS-induced AKT and JNK phosphorylation in primary microglial cells (n=3/group). **C.** Treatment with 1 μM Entrectinib and 10 μM Larotrectinib reduced LPS-stimulated p-p38 levels, whereas 1 μM Larotrectinib did not alter p38 phosphorylation (n=3/group). All values are presented as the mean ± SD. **p < 0.01, ****p < 0.0001 vs. control group; #p < 0.05, ###p < 0.001, ####p < 0.0001 vs. LPS group; comparisons indicated above connecting lines: ****p < 0.0001.

**
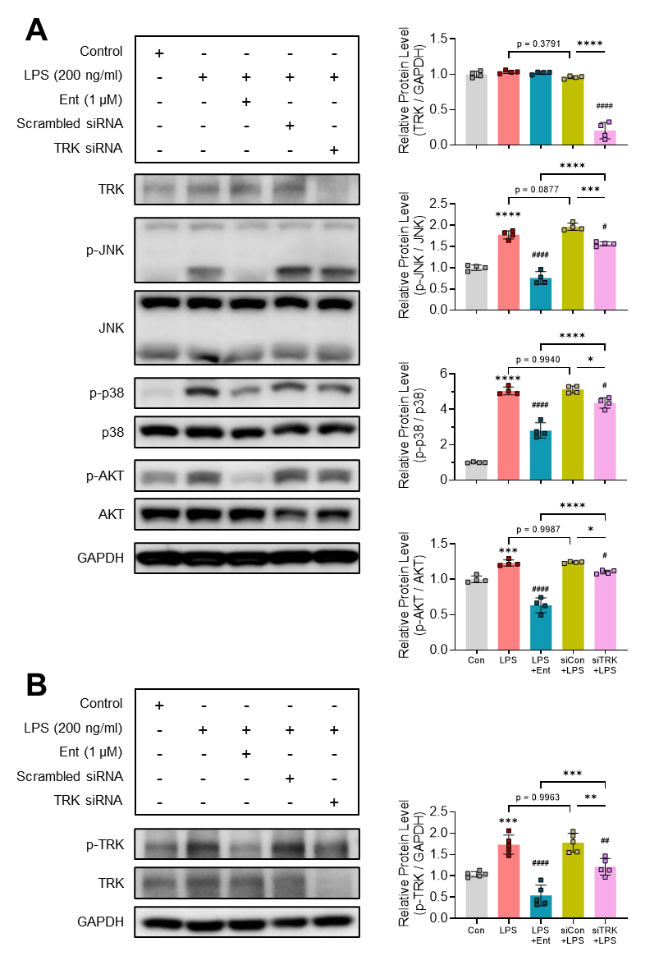
**

**Supplementary Figure 3.** **TRK knockdown partially recapitulates the inhibitory effects of Entrectinib on LPS-induced inflammatory signaling in primary microglia.** Primary microglial cells were transfected with scrambled siRNA (control) or siRNAs targeting TRK A/B, followed by LPS stimulation. **A.** Western blot analysis and quantitative densitometry of total TRK confirmed effective suppression of TRK expression following TRK siRNA transfection (n=4/group). In parallel, western blot analysis and quantification showed that LPS-induced phosphorylation of JNK, p38, and AKT was reduced following TRK knockdown, as well as after Entrectinib treatment (n = 4/group). **B.** Western blot analysis and quantification of phosphorylated TRK, normalized to GAPDH, demonstrated that Entrectinib treatment produced a greater reduction in TRK phosphorylation than TRK siRNA alone (n=5/group). All values are presented as the mean ± SD. ***p < 0.001, ****p < 0.0001 vs. control group; #p < 0.05, ##p < 0.01, ####p < 0.0001 vs. LPS group; comparisons indicated above connecting lines: *p < 0.05, **p < 0.01, ***p < 0.001, ****p < 0.0001.


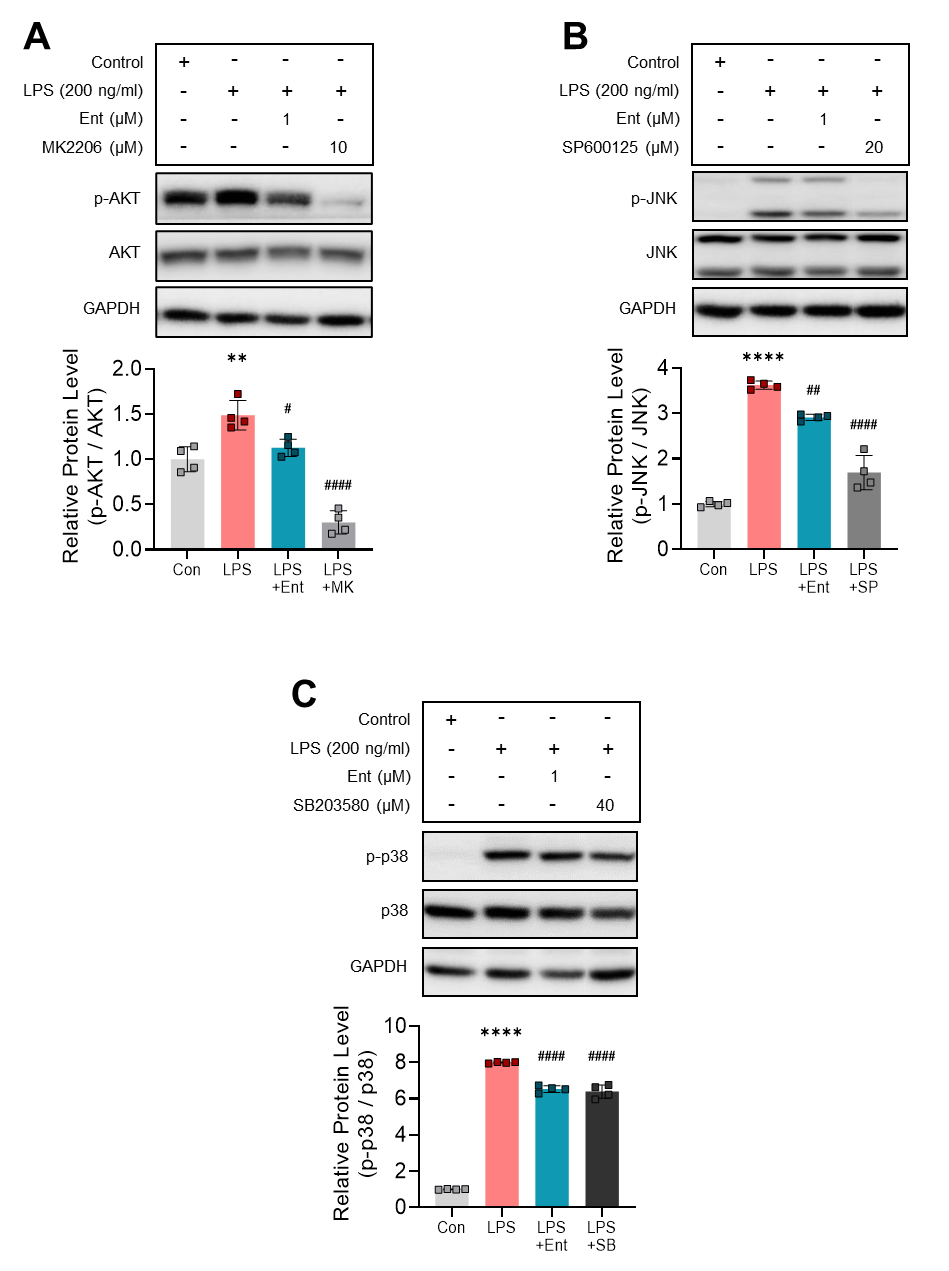


**Supplementary Figure 4. Entrectinib and selective inhibitors of AKT, JNK, and p38 suppress LPS-induced phosphorylation of AKT, JNK, and p38 in primary microglia. A-C.** Treatment with Entrectinib or the AKT inhibitor MK2206 (10 μM), JNK inhibitor SP600125 (20 μM), or p38 inhibitor SB203580 (40 μM) reduced LPS-induced phosphorylation of AKT, JNK, and p38 in primary microglia (n=4/group). All values are presented as the mean ± SD. **p < 0.01, ****p < 0.0001 vs. control group; ##p < 0.01, ###p < 0.001, ####p < 0.0001 vs. LPS group.


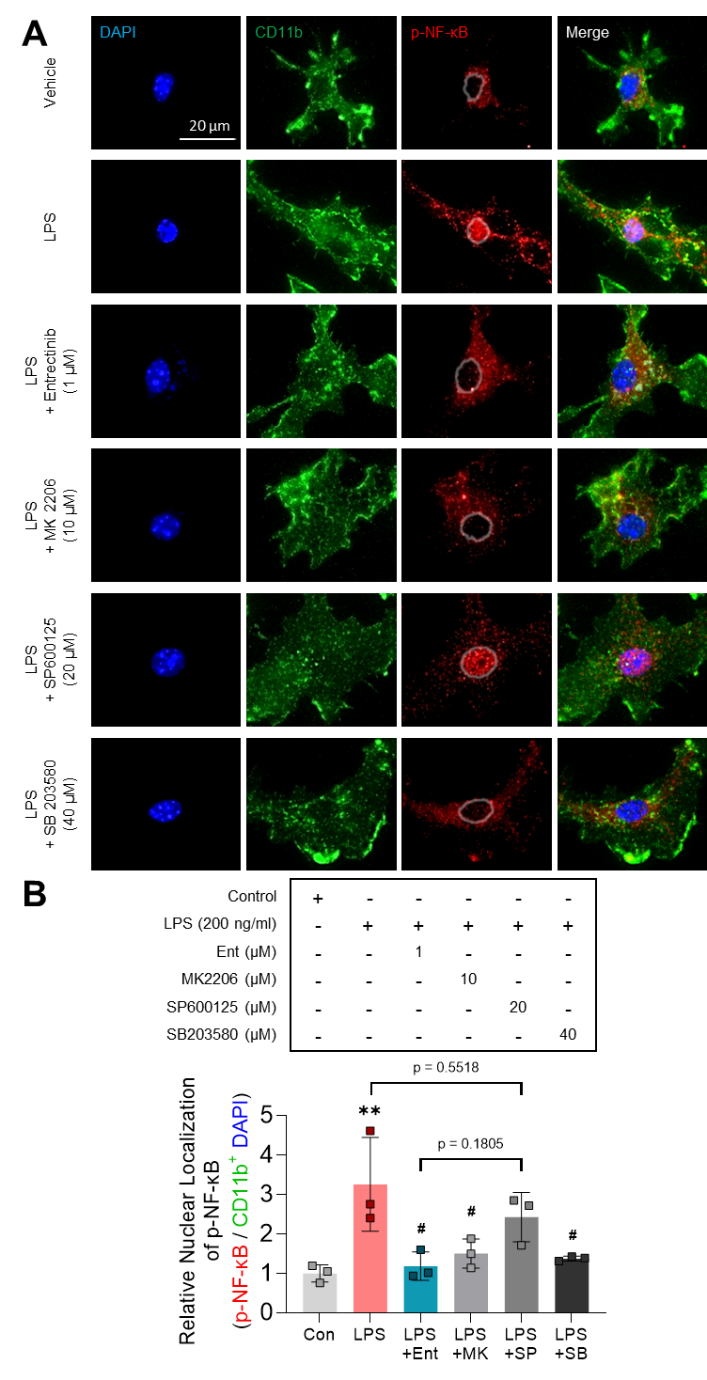


**Supplementary Figure 5. Entrectinib more effectively suppresses LPS-evoked nuclear translocation of p-NF-κB than Larotrectinib. A.** Immunocytochemistry of primary microglia using three markers: DAPI (nuclear marker, blue), CD11b (microglial marker, green), and p-NF-κB (red). White dashed circles indicate the nucleus of each cell. **B.** Quantitative analysis showed that Entrectinib, MK2206, and SB203580 reduced nuclear p-NF-κB fluorescence intensity following LPS treatment, whereas SP600125 did not alter nuclear p-NF-κB levels (n=3/group). All values are presented as the mean ± SD. **p < 0.01 vs. control group; #p < 0.05 vs. LPS group.


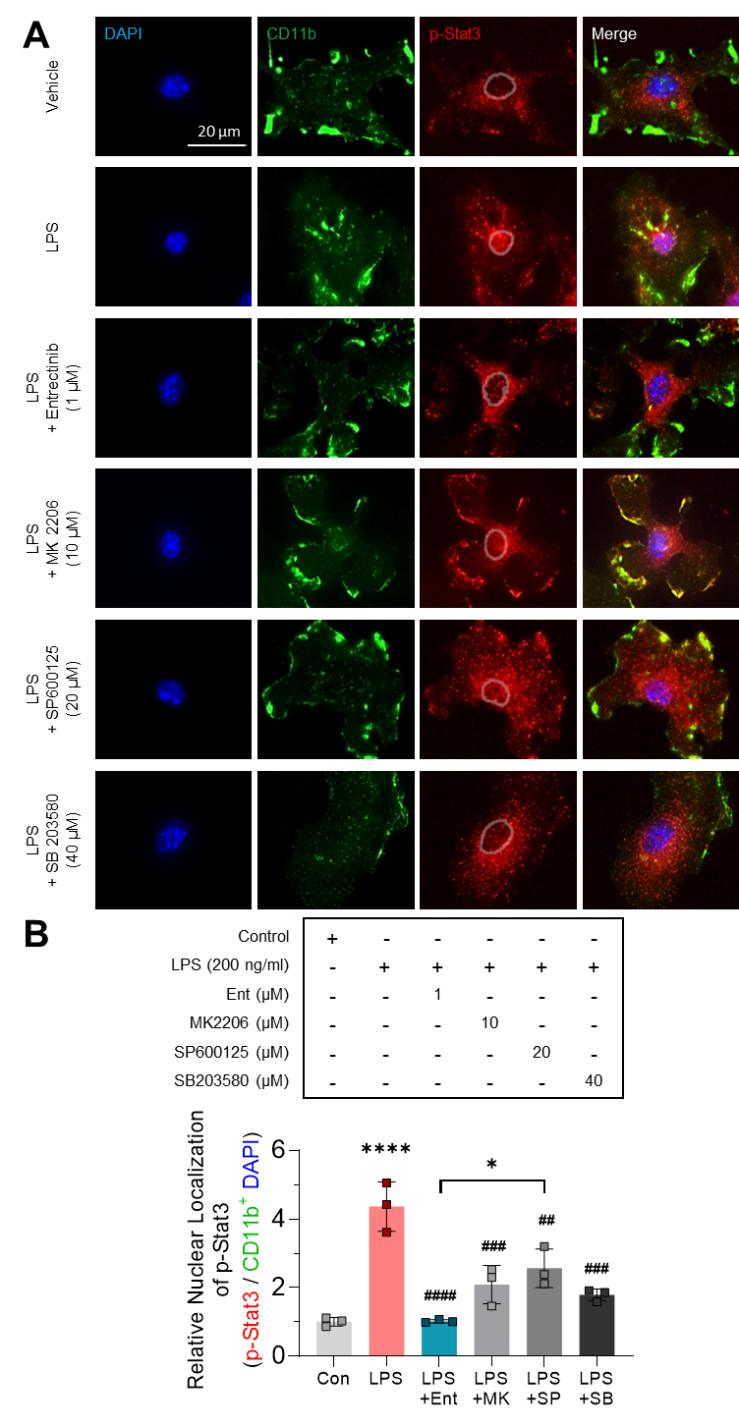


**Supplementary Figure 6. Entrectinib suppresses LPS-induced nuclear translocation of p-STAT3 more effectively than Larotrectinib. A.** Immunocytochemistry of primary microglia using three markers: DAPI (nuclear marker, blue), CD11b (microglial marker, green), and p-STAT3 (red). White dashed circles indicate the nucleus of each cell. **B.** Quantitative analysis showed that Entrectinib and pathway inhibitors reduced nuclear p-STAT3 fluorescence intensity following LPS treatment (n=3/group). All values are presented as the mean ± SD. ****p < 0.0001 vs. control group; ##p < 0.01, ###p < 0.001, ####p < 0.0001 vs. LPS group; comparisons indicated above connecting lines: *p < 0.05.

**
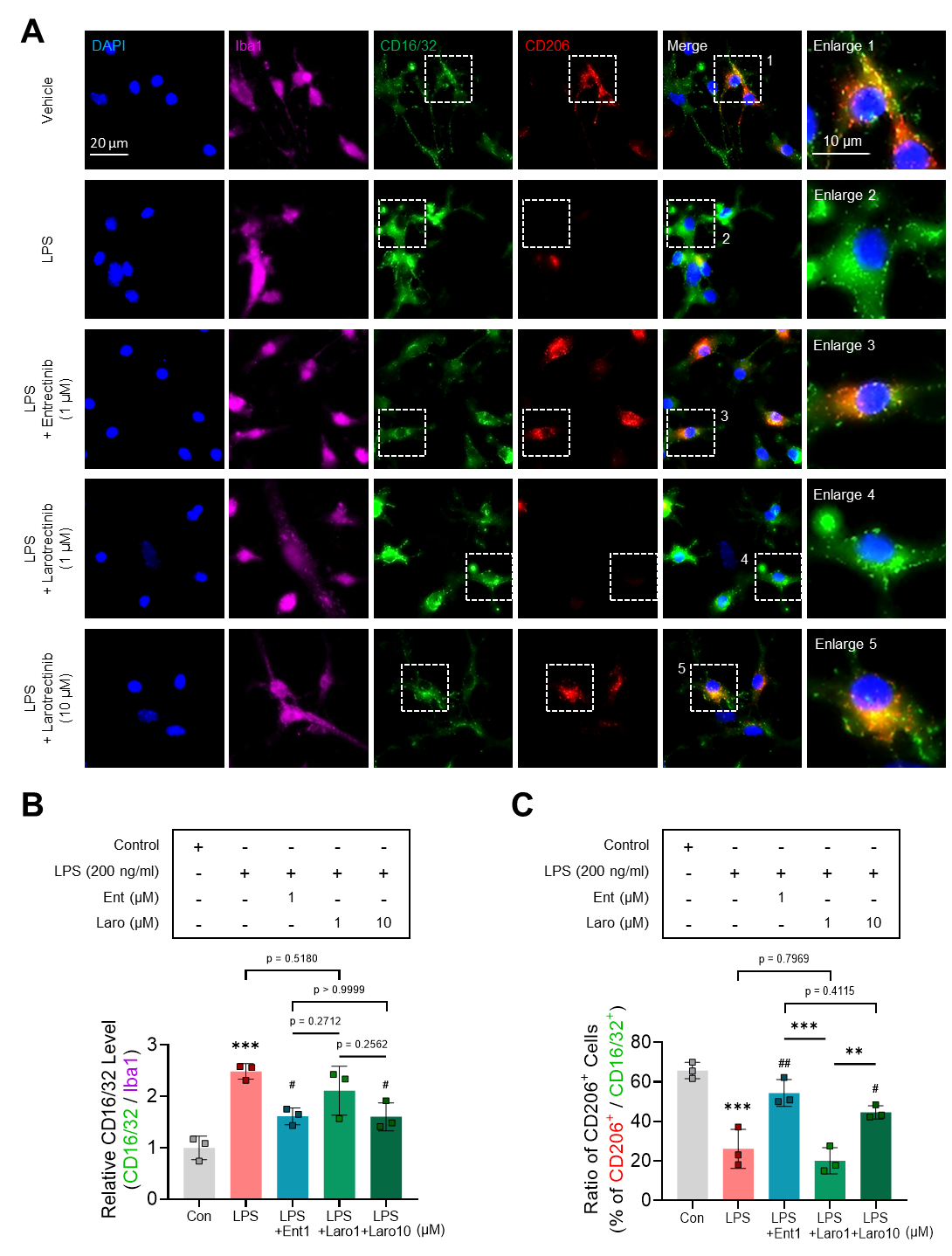
**

**Supplementary Figure 7. Entrectinib modulates LPS-induced changes in CD16/32 and CD206 expression more effectively than Larotrectinib. A.** Immunocytochemistry of primary microglia using four markers: DAPI (nuclear marker, blue), Iba-1 (microglial marker, magenta), CD16/32 (reactive microglial marker, green), and CD206 (anti-inflammatory microglial marker, red). White dashed rectangles indicate merged images shown at higher magnification. **B.** Quantitative analysis showed that treatment with 1 μM Entrectinib and 10 μM Larotrectinib reduced CD16/32 fluorescence intensity following LPS stimulation (n=3/group). **C.** CD206 fluorescence intensity increased following treatment with 1 μM Entrectinib and 10 μM Larotrectinib, whereas 1 μM Larotrectinib did not significantly alter CD206 expression (n=3/group). All values are presented as the mean ± SD. ***p < 0.001 vs. control group; #p < 0.05, ##p < 0.01 vs. LPS group; comparisons indicated above connecting lines: **p < 0.01, ***p < 0.001.


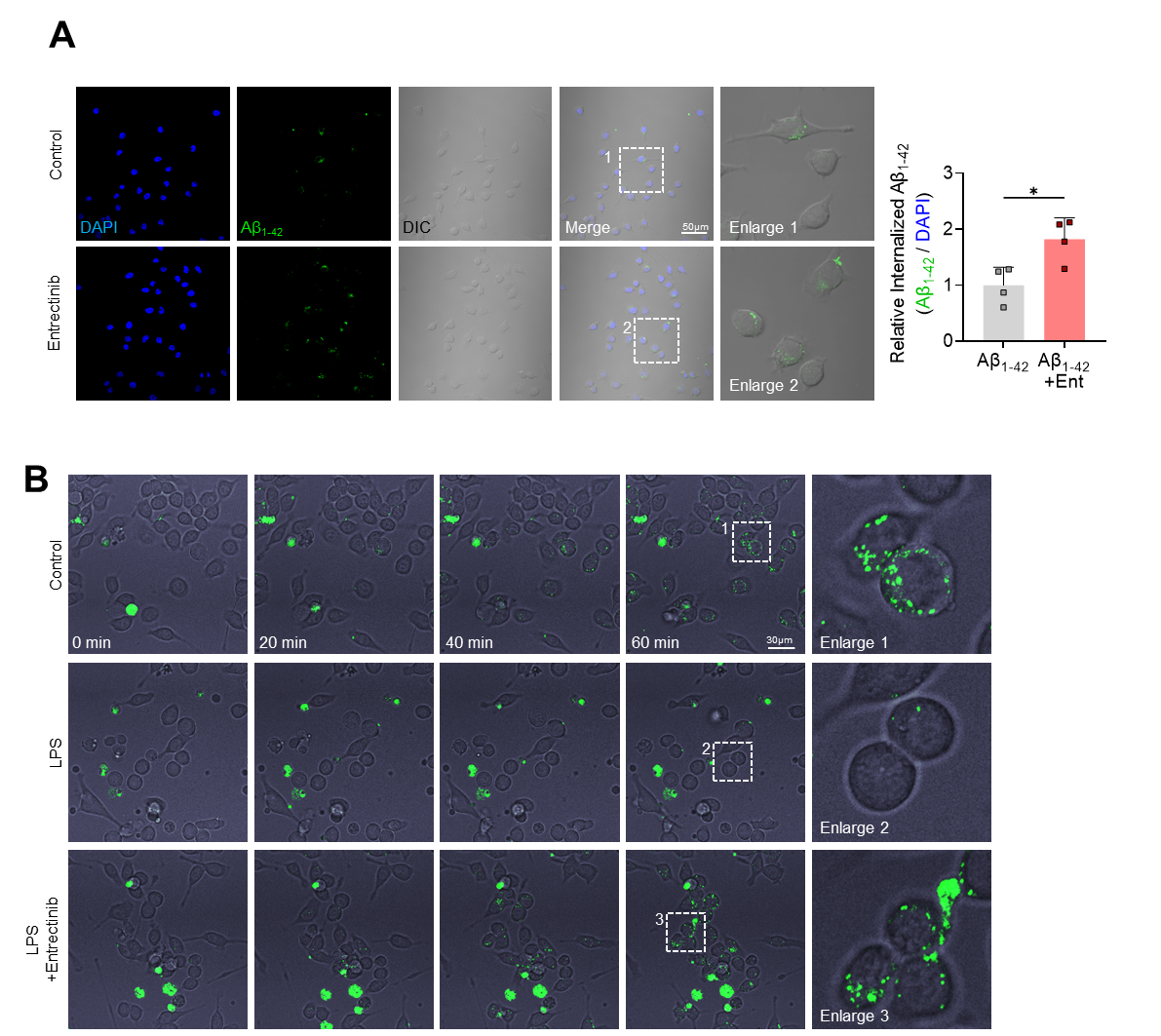


**Supplementary Figure 8. Entrectinib enhances phagocytic activity in BV2 microglial cells.** **A.** Fluorescence and DIC imaging showing microglial phagocytosis of Aβ following sequential treatment with Entrectinib and Aβ_1_–_42_–conjugated Alexa 488, as indicated (n=4/group). **B.** Live-cell imaging capturing microglial phagocytosis following sequential treatment with LPS, Entrectinib, and Aβ_1_–_42_-conjugated Alexa 488. Entrectinib increased intracellular Aβ_1_–_42_-conjugated Alexa 488 fluorescence intensity in BV2 microglial cells (n=4/group). White dashed rectangles indicate merged images shown at higher magnification. All values are presented as the mean ± SD. *p < 0.05.


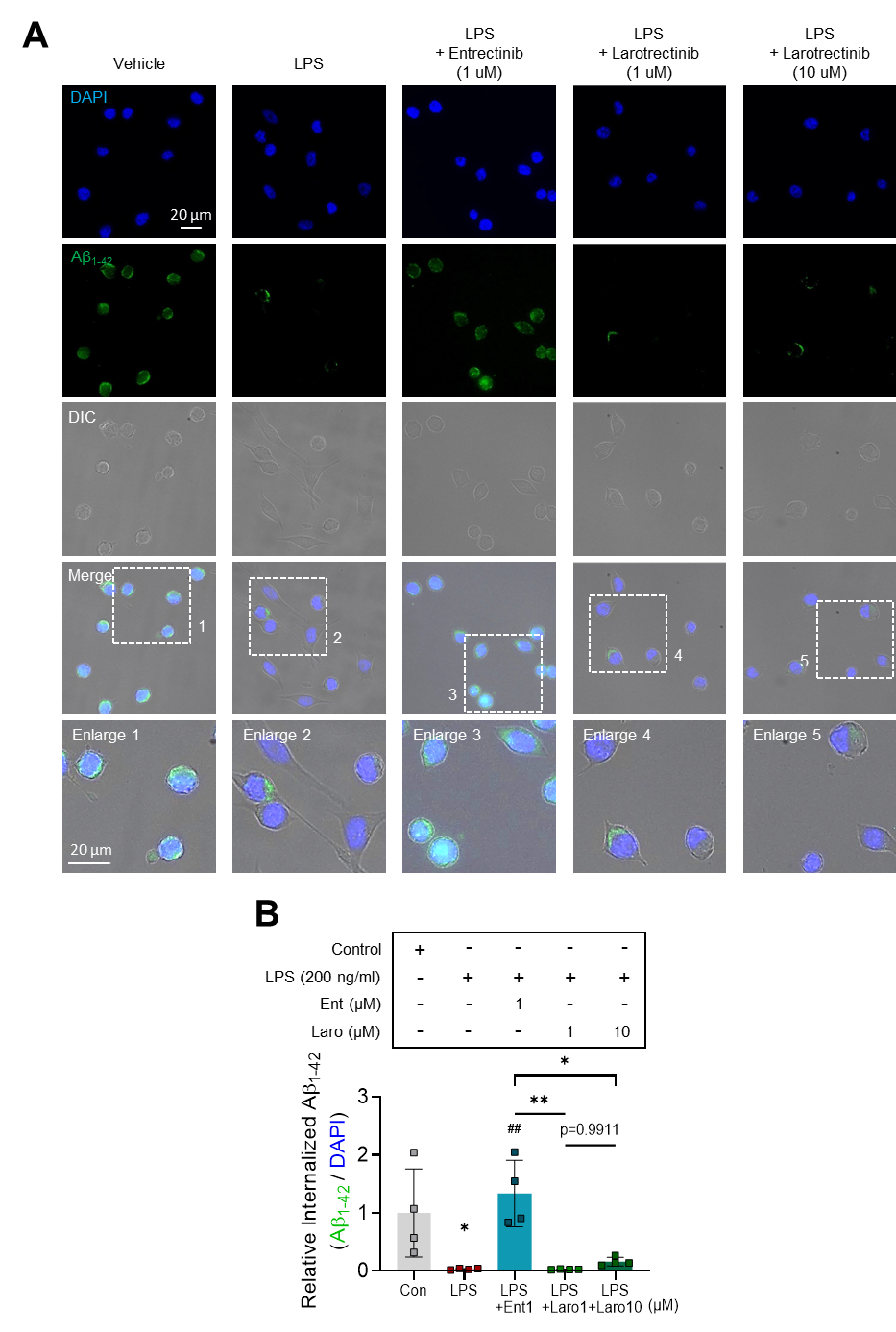


**Supplementary Figure 9. Entrectinib, but not Larotrectinib, enhances phagocytosis in BV2 microglial cells.** **A.** Fluorescence and DIC imaging showing microglial phagocytosis of Aβ following sequential treatment with Entrectinib or Larotrectinib and Aβ_1_–_42_-conjugated Alexa 488. White dashed rectangles indicate merged images shown at higher magnification. **B.** Quantitative analysis demonstrated that Entrectinib, but not Larotrectinib, increased intracellular Aβ_1_–_42_-conjugated Alexa 488 fluorescence intensity following LPS treatment (n=4/group). All values are presented as the mean ± SD. *p < 0.05 vs. control group; ##p < 0.01 vs. LPS group; comparisons indicated above connecting lines: *p < 0.05, **p < 0.01.

**
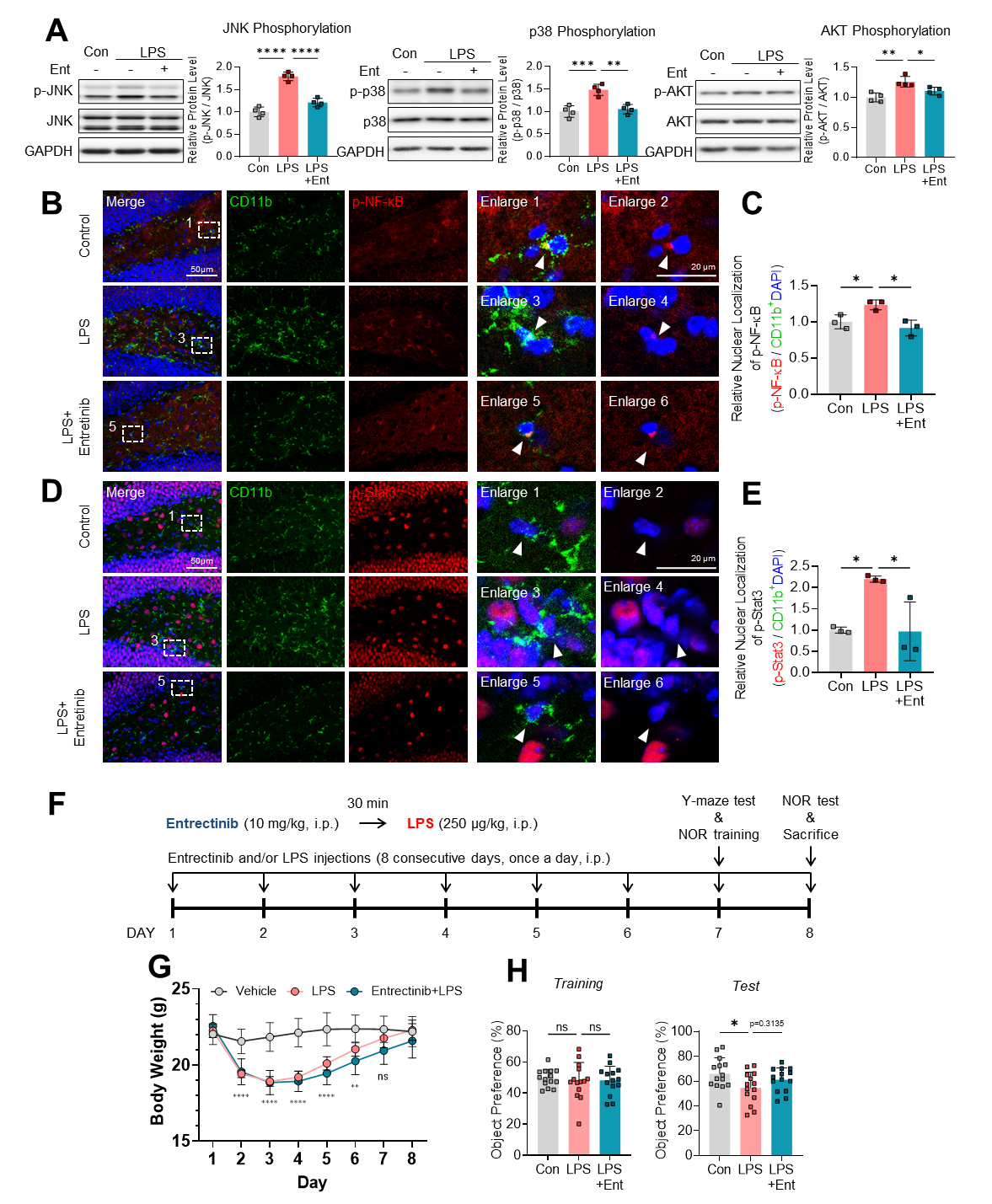
**

**Supplementary Figure 10. Entrectinib suppresses LPS-induced TRK-linked neuroinflammatory signaling and transcription factor activation in microglia. A.** Western blot analysis showing phosphorylation of JNK, p38, and AKT following LPS stimulation with Entrectinib pre-treatment in primary microglia. Entrectinib reduced LPS-induced phosphorylation of these signaling proteins (n=4/group). **B and D.** Immunofluorescence analysis showing nuclear translocation of microglial p-NF-κB and p-STAT3 in LPS-injected wild-type mice pre-treated with Entrectinib. White rectangles indicate regions shown at higher magnification; white arrows indicate representative cells. Enlarged panels (1, 3, and 5) show merged CD11b (green), DAPI (blue), and p-NF-κB or p-STAT3 (red) signals. Nuclear localization is highlighted in enlarged panels (2, 4, and 6). **C and E.** Quantitative analysis showing reduced nuclear p-NF-κB and p-STAT3 fluorescence intensity following Entrectinib treatment compared with LPS alone (n=3 mice/group, 4 slides/mouse; Control: 8–19 cells/slide; LPS: 21–41 cells/slide; LPS+Ent: 8–27 cells/slide). **F.** Schematic diagram of the behavioral testing paradigm following Entrectinib and LPS administration. **G.** Body weight changes recorded during 8 days of LPS and Entrectinib injections (n=14/group). **H.** NOR test used to assess memory performance (n=14/group). All values are presented as the mean ± SD. *p < 0.05, **p < 0.01, ***p < 0.001, ****p < 0.0001.

**
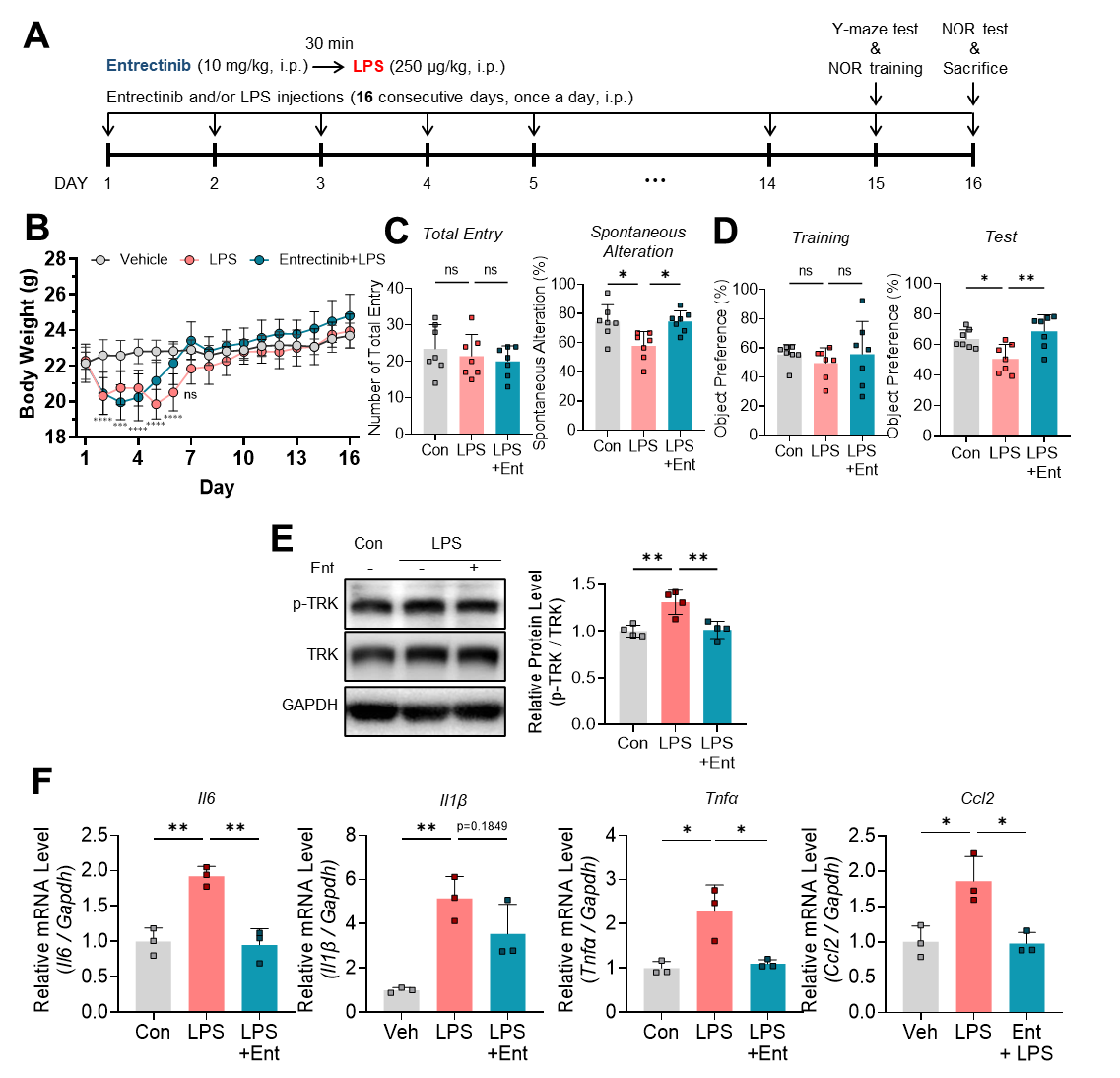
**

**Supplementary Figure 11. Long-term Entrectinib pre-treatment restores LPS-induced memory impairments, normalizes p-TRK levels, and suppresses proinflammatory factors in the hippocampus of mice. A.** Schematic diagram of the behavioral testing paradigm following Entrectinib pre-treatment and LPS administration. **B.** Body weight changes recorded during 16 days of LPS and Entrectinib injections (n=7/group). **C-D.** Y-maze and NOR tests were used to assess memory performance. Entrectinib pre-treatment mitigated LPS-induced reductions in spontaneous alternation and object preference in the Y-maze and NOR tests (n=7/group). **E.** Long-term Entrectinib pre-treatment reduced LPS-induced p-TRK protein levels in the hippocampus (n=4 mice/group). **F.** Real-time PCR analysis of hippocampal proinflammatory factors following sequential Entrectinib and LPS treatment, showing reduced cytokine expression after Entrectinib pre-treatment (n=3/group). All values are presented as the mean ± SD. *p < 0.05, **p < 0.01.


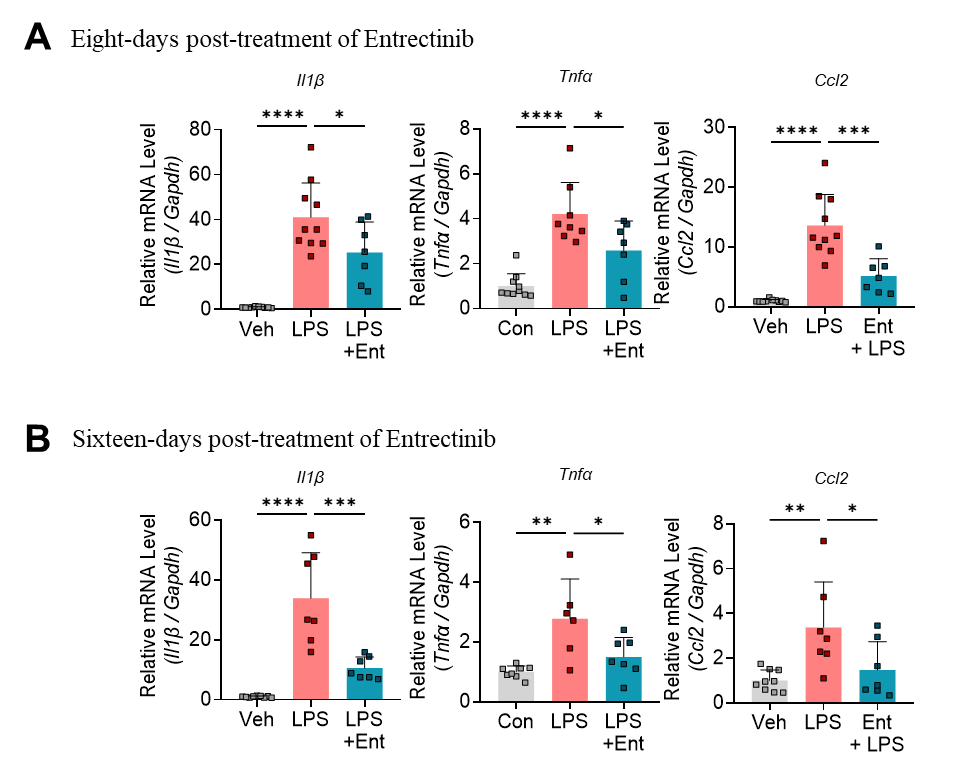


**Supplementary Figure 12. Short- and long-term Entrectinib post-treatment suppresses LPS-induced proinflammatory factor expression in the hippocampus. A.** Eight consecutive days of daily Entrectinib post-treatment reduced LPS-induced expression of the proinflammatory factors *Il1β, Tnfα,* and *Ccl2* in the hippocampus (n=7–10 mice/group). **B.** Sixteen consecutive days of daily Entrectinib post-treatment further attenuated LPS-induced expression of *Il1β, Tnfα,* and *Ccl2* in the hippocampus (n=6–10 mice/group). All values are presented as the mean ± SD. *p < 0.05, **p < 0.01, ***p < 0.001, ****p < 0.0001.

| **Gene** | **Forward Sequence (5’-3’)** | **Reverse Sequence (5’-3’)** |
| --- | --- | --- |
| ***mCcl2*** | GCTACAAGAGGATCACCAGCAG | GTCTGGACCCATTCCTTCTTGG |
| ***mCd33*** | GCATCTGATGCTGTGACTCCAG | AGTGTGGACACTGCTCTGTTCC |
| ***mCdc42*** | GATTGGTGGAGAGCCATACACTC | TGAGGATGGAGAGACCACTGAG |
| ***mCr2*** | AGAGTGTAAGCCAGTAGGACCAC | GACACAGTTGATAAGCACTCTCAC |
| ***mGapdh*** | TGATGGGTGTGAACCACGAG | TGATGGCATGGACTGTGGTC |
| ***mIl1β*** | ATGGCAACTGTTCCTGAACTCAACT | CAGGACAGGTATAGATTCTTTCCTTT |
| ***mIl13*** | CCTGGCTCTTGCTTGCCTT | GGTCTTGTGTGATGTTGCTCA |
| ***mIl23a*** | CATGCTAGCCTGGAACGCACAT | ACTGGCTGTTGTCCTTGAGTCC |
| ***mIl4*** | TGGGTCTCAACCCCCAGCTAGT | TGCATGGCGTCCCTTCTCCTGT |
| ***mIl6*** | TCCAGTTGCCTTCTTGGGAC | GTGTAATTAAGCCTCCGACTTG |
| ***mInos*** | ATGTCCGAAGCAAACATCAC | TAATGTCCAGGAAGTAGGTG |
| ***mSorl1*** | GAACACCTGTCTCCGAAACCAG | CGGAACTGAGTGTCTGCATCAC |
| ***mTnfα*** | TTCTGTCTACTGAACTTCGGGGTGATCGGTCC | GTATGAGATAGCAAATCGGCTGACGGTGTGGG |
| ***mTrem2*** | CTACCAGTGTCAGAGTCTCCGA | CCTCGAAACTCGATGACTCCTC |
| ***mVav1*** | ACAGTGCGTGAACGAGGTCAAG | GCCATAGTTAGCCAGAGACTGG |

**Supplementary Table 1**. List of primers used in real-time PCR for the present study.

| **Antibody** | **Host** | **Dilution**  **(WB/ICC or IF)** | **Cat no.**  **(Company)** |
| --- | --- | --- | --- |
| **anti-p-TRK** | Rabbit | 1:1000 (WB)  1:500 (ICC)  1:500 (IF) | 4621 (Cell Signaling Technology) |
| **anti-TRK** | Rabbit | 1:1000 (WB) | 929291 (Cell Signaling Technology) |
| **anti-p-JNK** | Rabbit | 1:1000 (WB)  1:100 (ICC) | 9251 (Cell Signaling Technology) |
| **anti-JNK** | Rabbit | 1:1000 (WB) | MBS8509129 (MyBioSource) |
| **anti-p-p38** | Rabbit | 1:1000 (WB)  1:100 (ICC) | 4511 (Cell Signaling Technology) |
| **anti-p38** | Rabbit | 1:1000 (WB) | 8690 (Cell Signaling Technology) |
| **anti-p-AKT** | Rabbit | 1:1000 (WB)  1:1000 (ICC) | 9271 (Cell Signaling Technology) |
| **anti-AKT** | Rabbit | 1:1000 (WB) | 9272 (Cell Signaling Technology) |
| **anti-p-NF-κB** | Rabbit | 1:1000 (WB)  1:100 (ICC)  1:500 (IF) | 3033 (Cell Signaling Technology) |
| **anti-NF-κB** | Rabbit | 1:1000 (WB) | 8242 (Cell Signaling Technology) |
| **anti-p-STAT3** | Rabbit | 1:1000 (WB)  1:100 (ICC)  1:500 (IF) | ab32143 (Abcam) |
| **anti-STAT3** | Rabbit | 1:1000 (WB) | ab68153 (Abcam) |
| **anti-Histone H3** | Rabbit | 1:10,000 (WB) | 9715 (Cell Signaling Technology) |
| **anti-GAPDH** | Rabbit | 1:10,000 (WB) | 2118 (Cell Signaling Technology) |
| **anti-PSD95** | Mouse | 1:1000 (WB) | 36233 (Cell Signaling Technology) |
| **anti-SYP** | Rabbit | 1:50,000 (WB) | 17785-1-AP (Proteintech) |
| **anti-CD11b** | Rat | 1:100 (ICC)  1:500 (IF) | 46512 (Cell Signaling Technology) |
| **anti-CD16/32** | Rat | 1:100 (ICC)  1:100 (IF) | 80366 (Cell Signaling Technology) |
| **anti-CD206** | Rabbit | 1:100 (ICC)  1:100 (IF) | 24595 (Cell Signaling Technology) |
| **anti-Iba1** | Chicken | 1:2000 (ICC) | PA5-143572 (Invitrogen) |

**Supplementary Table 2**. List of antibodies used in western blotting and immunostaining for the present study.
